# Quantitative proteomics reveals that distant recurrence-associated protein R-Ras and Transgelin predict post-surgical survival in patients with Stage III colorectal cancer

**DOI:** 10.18632/oncotarget.9701

**Published:** 2016-05-30

**Authors:** Lai Xu, Yanpan Gao, Yanyu Chen, Yi Xiao, Qingzhong He, Huizhong Qiu, Wei Ge

**Affiliations:** ^1^ Department of General Surgery, Peking Union Medical College Hospital, Chinese Academy of Medical Sciences and Peking Union Medical College, Beijing, 100730, China; ^2^ National Key Laboratory of Medical Molecular Biology & Department of Immunology, Institute of Basic Medical Sciences, Chinese Academy of Medical Sciences, Beijing, 100005, China

**Keywords:** quantitative proteomics, Stage III colorectal cancer, post-surgical recurrence, prognostic biomarker, R-Ras and Transgelin

## Abstract

Surgical resection supplemented with adjuvant chemotherapy is the current preferred treatment for Stage III colorectal cancer (CRC). However, as many as 48% of patients who undergo curative resection eventually suffer from incurable distant recurrence. To investigate the molecular mechanisms involved in Stage III CRC post-surgical distant recurrence, we identified a total of 146 differentially expressed proteins (DEPs) associated with distant recurrence in Stage III CRC using TMT-based quantitative mass spectrometry. Among these DEPs, the altered expressions of R-Ras and Transgelin were then validated in 192 individual specimens using immunohistochemistry (IHC). Furthermore, Kaplan-Meier analysis revealed that the levels of R-Ras and Transgelin were significantly associated with 5-year overall survival (OS) and disease-free survival (DFS), and multivariate Cox-regression analyses revealed that R-Ras and Transgelin were independent prognostic factors for OS and DFS, respectively. In conclusion, this study identified potential biochemical players involved in distant recurrence and indicates that R-Ras and Transgelin are potential post-surgical prognostic biomarkers for Stage III CRC. This proteomics data have been submitted to Proteome Xchange under accession number PXD002903.

## INTRODUCTION

Colorectal cancer (CRC) is a substantial health problem worldwide, with approximately 1,360,600 new cases diagnosed and 693,900 deaths in 2012, ranking second in newly-diagnosed cancer cases and fourth in cancer-related mortality [1]. Stage I and II CRC can be cured by surgical resection, while metastatic Stage IV is usually incurable [2]. For Stage III CRC, surgical resection with adjuvant chemotherapy is the standard of care [3]. Unfortunately, 48% of patients with Stage III CRC develop incurable distant recurrence within 5 years post-surgery [4]; this is one of the major obstacles to improving the prognosis of patients with CRC.

Several factors, such as nodal extension and tumor size [4], have been reported to be associated with the risk of distant recurrence in CRC patients. However, these factors provide little biochemical information of the primary tumor itself. To reveal the molecular features associated with post-surgical distant recurrence in patients with Stage III CRC, we used TMT-based quantitative mass spectrometry to investigate the proteomic difference between the tumor tissues of patients with a good outcome and patients who suffered from distant recurrence. A total of 146 differentially expressed proteins (DEPs) were identified and over-representation of Gene Ontology (GO) categories, biological pathways and protein complexes within these DEPs were assessed using bioinformatics tools. The results revealed that the proteins related to extracellular matrix, exosome and contractile fiber play an important role in the tumor relapse. Among the 146 DEPs, R-Ras and Transgelin were further validated via immunohistochemistry (IHC) and clinicopathological statistics, and the expression levels of these proteins were found to correlate positively with the survival outcome of Stage III CRC patients. This study not only provides an insight into the cellular and molecular mechanisms involved in the post-surgical distant recurrence, but also reveals that R-Ras and Transgelin may serve as prognostic biomarkers of Stage III CRC in clinical practice.

## RESULTS

### TMT-based quantitative MS identified 146 DEPs associated with post-surgical distant recurrence in patients with Stage III CRC

Based on the depth of tumor growth and the number of positive regional lymph nodes, Stage III CRC is subdivided into IIIA, IIIB and IIIC in the TNM Staging System [5, 6]. Stage IIIA is much less common than IIIB and IIIC CRC, and notably, has a relatively good prognosis [5, 6]. At the beginning of this study, we compared protein abundances in the tumor tissues of patients with a good outcome and patients who suffered distant recurrence in a high-throughput manner using two TMT-based quantitative MS experiments (Table 1). Stage IIIB and IIIC specimens were respectively recruited in order to explore the subgroup-specific factors that potentially influence post-surgical distant recurrence in Stage III CRC.

**Table 1:** Clinicopathological information of patients recruited in the MS experiments

Experiment No.	Patient ID	TMT Label	AJCC Stage	TNM Stage	Survival (month)	DFI (month)	First Recurrence Site	Age (year)	Gender	Tumor differentiation	Adjuvant Treatment
**1**	G1	126	IIIB	T4aN1bM0	75	75	--	67	M	Moderate	Mayo Clinic
	G2	127	IIIB	T3N1aM0	74	74	--	63	F	Moderate	FOLFOX4
	P1	128	IIIB	T4aN1bM0	32	13	Liver	53	M	Well	FOLFOX4
	P2	130	IIIB	T3N1aM0	35	26	Lung	70	F	Poor	FOLFOX4
**2**	G3	126	IIIC	T4aN2aM0	70	70	--	68	F	Moderate	FOLFOX4
	G4	128	IIIC	T4aN2aM0	66	66	--	57	M	Well	FOLFOX4
	P3	130	IIIC	T4aN2aM0	51	22	Lung	55	M	Moderate	FOLFOX4
	P4	131	IIIC	T4aN2bM0	13	10	Liver/Bone	71	M	Moderate	XELOX

(Mayo Clinic regimen: 5-fluorouracil and leucovorin FOLFOX4 regimen: 5-fluorouracil, leucovorin and oxaliplatin.

XELOX regimen: capecitabine and oxaliplatin. Patient survival was defined as living time lasting from diagnosis of CRC to death or last follow-up. DFI was censored if the patient remained tumor recurrence free at the time of death or at the last follow-up)

DFI: disease-free interval.

Each MS experiment analyzed two patients with a good outcome and two patients who developed post-surgical distant recurrence (Table 1). The patient tumor specimens were homogenized, solubilized, digested and then labeled with isobaric TMT reagents of isotopic reporters. Consequently, the labeled samples were pooled and analyzed via MS, and the raw spectrum data were analyzed using Proteome Discoverer 1.4. Eventually, 3,222 and 2,818 proteins were identified in the Stage IIIB and IIIC patient groups, respectively (Supplementary Table S1) with an overlap of 2,383 (>73.9%) proteins (Supplementary Figure S1A). More than 99.2% (3,198 of 3,222 and 2,798 of 2,818) of the proteins in each group were quantifiable.

Based on the criteria given in the “Data analysis” part of MATERIALS AND METHODS, a total of 146 distant recurrence-associated DEPs were selected from the Stage IIIB and IIIC groups (Supplementary Figure S1B); the relative abundance of these proteins is listed in Table 2. In the Stage IIIB group, 41 proteins were upregulated and 88 proteins were downregulated in patients who developed distant recurrence. In the Stage IIIC group, 13 proteins were upregulated and 8 proteins were downregulated in patients who developed distant recurrence. More than 50% of the DEPs exhibited a protein score greater than 10 (Supplementary Figure S1B). Four proteins were differentially expressed in both the Stage IIIB and IIIC groups: MYH11, DES and CEP131 were downregulated in patients who suffered distant recurrence in both Stage IIIB and IIIC groups, while SDF2L was downregulated in Stage IIIB but upregulated in IIIC.

**Table 2:** Overview of the 146 DEPs

Accession	Description	Score		Coverage (%)		Relative Abundance in IIIB Group				Relative Abundance in IIIC Group				TMT Ratio Variability in IIIB Group			TMT Ratio Variability in IIIC Group			MW (Da)
		IIIB	IIIC	IIIB	IIIC	G1	G2	P1	P2	G3	G4	P3	P4	G2/G1	P1/G1	P2/G1	G4/G3	P3/G3	P4/G3	
P05109	Protein S100-A8 GN=S100A8	241.2	271.8	48.4	53.8	1.0	0.9	1.5	1.6	1.0	1.7	1.1	1.5	9.2	9.9	8.3	24.5	22.9	16.8	10.8
P31943	Heterogeneous nuclear ribonucleoprotein H GN=HNRNPH1	165.0	200.7	32.3	27.6	1.0	1.1	1.8	1.9	1.0	0.7	0.7	1.1	12.8	82.7	39.8	24.4	48.4	10.6	49.2
P09429	High mobility group protein B1 GN=HMGB1	159.6	59.0	32.1	25.1	1.0	0.9	2.1	1.7	1.0	0.4	0.7	0.6	15.0	37.5	26.4	46.6	8.9	26.4	24.9
P02788	Lactotransferrin GN=LTF	155.8	302.1	34.7	45.1	1.0	0.9	1.9	1.6	1.0	2.1	1.2	1.6	13.1	41.8	41.2	47.4	19.5	21.3	78.1
P59666	Neutrophil defensin 3 GN=DEFA3	112.5	ND	27.7	ND	1.0	0.9	1.8	1.6	ND	ND	ND	ND	18.3	25.3	23.9	ND	ND	ND	10.8
P06731	Carcinoembryonic antigen-related cell adhesion molecule 5 GN=CEACAM5	47.7	26.8	5.1	6.4	1.0	0.9	1.6	1.8	1.0	1.2	1.7	4.3	17.7	51.5	118.4	25.2	65.5	76.2	76.7
P55060	Exportin-2 GN=CSE1L	42.4	9.6	5.5	5.5	1.0	1.1	1.9	2.1	1.0	0.8	0.7	1.2	22.0	74.6	55.8	8.0	18.7	7.9	110.3
P11387	DNA topoisomerase 1 GN=TOP1	33.8	33.2	8.8	7.3	1.0	0.9	1.5	1.6	1.0	0.8	0.9	1.3	9.5	8.1	18.0	20.7	17.8	10.8	90.7
P55209	Nucleosome assembly protein 1-like 1 GN=NAP1L1	32.6	41.9	7.4	11.8	1.0	1.3	2.0	3.6	1.0	0.5	0.7	0.7	17.1	24.1	50.1	52.3	3.6	20.2	45.3
Q9UNH7	Sorting nexin-6 GN=SNX6	31.7	20.3	13.3	14.0	1.0	1.2	2.0	1.9	1.0	0.7	1.7	1.1	15.6	18.0	18.6	38.8	37.3	4.9	46.6
P26583	High mobility group protein B2 GN=HMGB2	30.2	16.7	18.7	18.2	1.0	1.0	2.3	1.9	1.0	0.5	0.7	0.6	0.8	11.6	22.2	31.9	50.0	19.9	24.0
P42167	Lamina-associated polypeptide 2, isoforms beta/gamma GN=TMPO	29.6	ND	15.0	ND	1.0	1.0	2.0	1.8	ND	ND	ND	ND	ND	ND	ND	ND	ND	ND	ND
Q9Y2×3	Nucleolar protein 58 GN=NOP58	26.3	29.7	4.9	7.9	1.0	1.0	1.7	1.6	1.0	0.6	0.8	1.3	7.7	17.8	27.2	36.8	9.2	9.4	59.5
Q9NX24	H/ACA ribonucleoprotein complex subunit 2 GN=NHP2	22.9	24.4	24.8	31.4	1.0	1.2	2.4	2.0	1.0	0.8	0.9	1.0	7.6	100.8	25.3	32.9	6.0	23.3	17.2
P22087	rRNA 2′-O-methyltransferase fibrillarin GN=FBL	22.7	15.3	20.3	19.9	1.0	1.0	1.8	1.7	1.0	0.6	0.7	1.2	8.6	17.5	46.7	12.6	10.4	9.6	33.8
P67809	Nuclease-sensitive element-binding protein 1 GN=YBX1	19.5	47.2	17.3	11.1	1.0	1.0	1.9	1.9	1.0	0.4	0.6	0.5	9.8	54.8	70.9	59.5	37.7	46.6	35.9
P51531	Probable global transcription activator SNF2L2 GN=SMARCA2	18.6	ND	2.9	ND	1.0	1.0	2.6	1.7	ND	ND	ND	ND	35.7	3.2	3.5	ND	ND	ND	10.8
P43487	Ran-specific GTPase-activating protein GN=RANBP1	16.9	15.7	11.0	11.0	1.0	1.1	2.1	2.2	1.0	0.7	0.8	1.1	24.9	43.3	41.1	5.4	8.3	7.8	23.3
Q9Y3A5	Ribosome maturation protein SBDS GN=SBDS	16.7	7.0	14.4	7.2	1.0	1.1	1.7	1.7	1.0	0.5	0.7	0.8	11.1	13.4	9.0	ND	ND	ND	28.7
Q96GG9	DCN1-like protein 1 GN=DCUN1D1	12.8	16.4	11.2	11.2	1.0	0.9	1.6	1.5	1.0	0.6	0.8	1.1	3.8	16.5	20.6	28.5	21.6	3.5	30.1
Q6WKZ4	Rab11 family-interacting protein 1 GN=RAB11FIP1	12.0	4.9	1.6	1.6	1.0	1.1	2.1	2.0	1.0	0.8	1.0	1.4	10.1	31.9	19.6	23.0	6.8	19.5	137.1
P62633	Cellular nucleic acid-binding protein GN=CNBP	11.2	17.4	7.9	7.9	1.0	1.3	3.1	2.1	1.0	0.5	0.4	0.7	9.3	14.8	18.8	8.2	12.2	5.8	19.4
P51116	Fragile X mental retardation syndrome-related protein 2 GN=FXR2	10.5	2.3	2.7	1.5	1.0	1.0	1.6	1.6	1.0	0.7	0.9	1.2	6.6	15.6	15.1	ND	ND	ND	74.2
O14602	Eukaryotic translation initiation factor 1A, Y-chromosomal GN=EIF1AY	8.5	ND	6.9	ND	1.0	1.2	2.4	2.1	ND	ND	ND	ND	2.9	62.7	0.4	ND	ND	ND	10.8
Q9NTI5	Sister chromatid cohesion protein PDS5 homolog B GN=PDS5B	8.0	6.2	2.0	2.0	1.0	1.1	1.8	2.3	1.0	0.8	0.7	1.3	12.7	24.9	18.4	4.0	11.4	4.3	164.6
Q9H307	Pinin GN=PNN	7.9	ND	3.1	ND	1.0	1.3	2.6	3.2	ND	ND	ND	ND	21.3	24.5	18.7	ND	ND	ND	10.8
Q9BSD7	Cancer-related nucleoside-triphosphatase GN=NTPCR	7.3	ND	7.9	ND	1.0	1.0	2.1	1.7	ND	ND	ND	ND	11.1	16.3	0.8	ND	ND	ND	49.2
O60216	Double-strand-break repair protein rad21 homolog GN=RAD21	6.7	ND	4.0	ND	1.0	1.0	2.1	1.6	ND	ND	ND	ND	6.9	4.5	51.7	ND	ND	ND	24.9
P16112	Aggrecan core protein GN=ACAN	6.6	ND	0.6	ND	1.0	1.0	2.4	2.1	ND	ND	ND	ND	3.9	7.6	14.6	ND	ND	ND	78.1
O60869	Endothelial differentiation-related factor 1 GN=EDF1	6.5	3.3	10.1	10.1	1.0	1.2	2.4	2.9	1.0	0.4	0.5	0.6	44.1	30.2	59.7	ND	ND	D	16.4
P80511	Protein S100-A12 GN=S100A12	6.4	20.2	9.8	9.8	1.0	0.7	7.7	1.7	1.0	5.9	0.6	2.4	5.8	13.1	0.2	10.4	41.8	11.2	10.6
Q13043	Serine/threonine-protein kinase 4 GN=STK4	3.4	ND	2.3	ND	1.0	1.0	1.8	1.6	ND	ND	ND	ND	ND	ND	ND	ND	ND	ND	10.8
O00483	Cytochrome c oxidase subunit NDUFA4 GN=NDUFA4	3.2	8.2	9.9	19.8	1.0	1.1	2.2	2.0	1.0	0.9	1.1	1.0	ND	ND	ND	8.1	4.0	5.3	9.4
P21741	Midkine GN=MDK	2.9	2.8	7.0	7.0	1.0	0.9	1.7	1.9	1.0	0.6	1.1	0.7	ND	ND	ND	ND	ND	ND	15.6
P00403	Cytochrome c oxidase subunit 2 GN=MT-CO2	2.6	ND	4.4	ND	1.0	0.8	1.6	1.6	ND	ND	ND	ND	ND	ND	ND	ND	ND	ND	10.8
Q99614	Tetratricopeptide repeat protein 1 GN=TTC1	2.6	ND	4.5	ND	1.0	1.1	2.1	1.7	ND	ND	ND	ND	ND	ND	ND	ND	ND	ND	49.2
Q2TB90	Putative hexokinase HKDC1 GN=HKDC1	2.4	ND	1.5	ND	1.0	1.0	2.4	1.9	ND	ND	ND	ND	ND	ND	ND	ND	ND	ND	24.9
O14653	Golgi SNAP receptor complex member 2 GN=GOSR2	2.1	ND	3.8	ND	1.0	1.0	1.5	1.6	ND	ND	ND	ND	ND	ND	ND	ND	ND	ND	78.1
Q6P158	Putative ATP-dependent RNA helicase DHX57 GN=DHX57	2.0	0.0	0.8	0.8	1.0	1.1	1.9	1.7	1.0	0.6	0.7	0.8	ND	ND	ND	ND	ND	ND	155.5
Q96CU9	FAD-dependent oxidoreductase domain-containing protein 1 GN=FOXRED1	1.8	ND	2.3	ND	1.0	1.0	1.5	1.5	ND	ND	ND	ND	ND	ND	ND	ND	ND	ND	10.8
A0PJK1	Sodium/glucose cotransporter 5 GN=SLC5A10	1.8	ND	2.4	ND	1.0	1.0	2.5	2.0	ND	ND	ND	ND	ND	ND	ND	ND	ND	ND	49.2
P35749	Myosin-11 GN=MYH11	1542.6	2869.6	45.6	52.7	1.0	0.9	0.5	0.5	1.0	1.2	0.6	0.6	11.0	47.4	35.6	10.6	79.2	41.4	227.2
P17661	Desmin GN=DES	917.8	2834.1	58.9	71.7	1.0	1.1	0.3	0.3	1.0	0.9	0.5	0.5	8.9	101.6	57.4	11.4	63.6	43.4	53.5
P01877	Ig alpha-2 chain C region GN=IGHA2	622.4	285.9	61.2	31.5	1.0	0.7	0.3	0.3	1.0	2.0	1.1	2.4	9.3	29.2	26.0	32.3	19.8	28.1	36.5
P12277	Creatine kinase B-type GN=CKB	470.8	174.1	48.6	38.3	1.0	1.3	0.5	0.6	1.0	1.6	0.8	1.3	21.9	28.9	17.8	25.2	45.0	18.4	42.6
Q9Y6R7	IgGFc-binding protein GN=FCGBP	454.4	266.0	15.5	11.5	1.0	0.9	0.5	0.4	1.0	2.2	1.3	2.6	12.9	72.2	57.8	83.3	26.3	60.4	571.6
Q05707	Collagen alpha-1(XIV) chain GN=COL14A1	382.6	405.8	19.2	22.6	1.0	0.9	0.5	0.4	1.0	1.3	2.1	1.2	10.5	32.1	41.6	22.0	35.3	13.7	193.4
P24844	Myosin regulatory light polypeptide 9 GN=MYL9	256.7	536.8	59.9	66.3	1.0	1.0	0.6	0.6	1.0	1.2	0.7	0.7	23.2	36.6	27.3	12.4	60.9	47.4	19.8
Q14315	Filamin-C GN=FLNC	216.0	401.0	14.9	21.6	1.0	0.9	0.6	0.6	1.0	0.9	0.7	0.7	11.5	24.8	26.7	11.1	34.9	42.4	290.8
Q02817	Mucin-2 GN=MUC2	211.1	79.3	6.1	2.9	1.0	1.1	0.4	0.5	1.0	2.4	1.4	3.6	36.3	80.2	47.4	60.1	27.6	72.1	540.0
Q13228	Selenium-binding protein 1 GN=SELENBP1	208.6	174.3	39.6	36.9	1.0	1.4	0.4	0.5	1.0	1.6	1.5	1.8	18.5	50.2	45.4	40.4	34.0	36.9	52.4
P00915	Carbonic anhydrase 1 GN=CA1	191.5	94.7	40.6	24.1	1.0	1.2	0.5	0.5	1.0	1.1	1.5	1.0	19.4	42.9	29.9	14.4	44.9	15.5	28.9
O95994	Anterior gradient protein 2 homolog GN=AGR2	182.4	199.9	50.3	46.9	1.0	1.0	0.5	0.6	1.0	0.9	0.7	1.7	10.7	60.7	52.8	10.0	57.0	13.9	20.0
P01833	Polymeric immunoglobulin receptor GN=PIGR	181.2	169.7	24.2	18.5	1.0	0.8	0.3	0.3	1.0	2.0	2.0	4.4	10.8	78.0	45.3	47.8	37.0	76.8	83.2
Q8WWA0	Intelectin-1 GN=ITLN1	106.1	29.9	32.0	15.7	1.0	0.5	0.3	0.3	1.0	2.7	1.3	3.5	25.8	138.0	88.2	2.3	10.9	19.6	34.9
P01871	Ig mu chain C region GN=IGHM	83.1	62.0	25.2	25.9	1.0	0.9	0.5	0.5	1.0	1.6	1.5	1.1	9.8	37.1	41.1	10.5	20.9	11.5	49.3
Q9NR45	Sialic acid synthase GN=NANS	78.8	42.5	24.8	14.2	1.0	1.1	0.6	0.6	1.0	1.3	1.0	1.7	11.8	39.7	43.7	19.4	22.1	25.1	40.3
P00326	Alcohol dehydrogenase 1C GN=ADH1C	78.3	28.6	24.3	19.7	1.0	0.8	0.4	0.4	1.0	3.4	2.6	3.3	13.8	22.8	15.2	53.8	25.4	43.0	39.8
Q15661	Tryptase alpha/beta-1 GN=TPSAB1	73.4	114.3	24.4	26.6	1.0	0.9	0.4	0.5	1.0	1.9	1.6	1.8	6.1	55.5	33.8	49.3	32.1	29.9	30.5
P00325	Alcohol dehydrogenase 1B GN=ADH1B	70.6	32.5	21.9	21.1	1.0	0.9	0.5	0.5	1.0	1.1	1.4	1.3	1.0	44.3	45.2	1.3	16.8	1.4	39.8
Q07654	Trefoil factor 3 GN=TFF3	69.0	19.3	47.5	47.5	1.0	0.9	0.5	0.5	1.0	2.0	1.3	2.6	7.9	29.2	22.2	11.5	8.9	21.2	8.6
P00918	Carbonic anhydrase 2 GN=CA2	64.6	44.0	49.6	33.1	1.0	1.7	0.5	0.6	1.0	1.5	1.7	1.5	17.6	29.6	20.1	35.2	16.7	16.3	29.2
P55268	Laminin subunit beta-2 GN=LAMB2	60.6	81.0	9.3	14.1	1.0	1.1	0.6	0.6	1.0	1.3	1.4	1.2	15.4	36.9	18.1	9.9	15.3	15.3	195.9
Q96BQ1	Protein FAM3D GN=FAM3D	46.2	ND	29.0	ND	1.0	1.0	0.3	0.4	ND	ND	ND	ND	4.1	36.2	34.8	ND	ND	ND	10.8
P28799	Granulins GN=GRN	41.6	72.8	11.3	17.7	1.0	0.9	0.6	0.5	1.0	1.4	1.4	1.7	16.6	27.9	25.5	27.3	40.3	22.4	63.5
Q13642	Four and a half LIM domains protein 1 GN=FHL1	39.1	30.7	15.2	15.2	1.0	1.1	0.6	0.6	1.0	0.8	1.1	0.7	11.4	14.1	30.1	31.7	55.1	47.5	36.2
P30049	ATP synthase subunit delta, mitochondrial GN=ATP5D	36.8	5.8	17.3	5.4	1.0	0.9	0.6	0.5	1.0	1.0	1.0	1.5	10.4	16.4	18.1	11.8	6.1	9.5	17.5
Q15124	Phosphoglucomutase-like protein 5 GN=PGM5	36.4	42.3	8.6	8.3	1.0	0.8	0.4	0.4	1.0	1.1	0.9	0.9	12.4	53.1	59.1	18.4	64.4	48.9	62.2
Q9HCY8	Protein S100-A14 GN=S100A14	36.3	25.1	52.9	25.0	1.0	0.9	0.5	0.5	1.0	0.7	0.5	0.9	14.0	19.6	34.9	31.9	96.0	15.7	11.7
Q96C23	Aldose 1-epimerase GN=GALM	33.6	17.3	8.5	8.5	1.0	1.1	0.5	0.6	1.0	1.2	1.1	1.5	8.5	15.2	13.1	7.8	25.0	9.2	37.7
P25774	Cathepsin S GN=CTSS	33.2	30.6	16.6	20.9	1.0	1.0	0.6	0.7	1.0	1.3	1.5	1.8	5.0	26.2	9.8	22.4	16.0	24.8	37.5
P01591	Immunoglobulin J chain GN=IGJ	32.7	16.0	22.6	7.6	1.0	0.8	0.5	0.5	1.0	3.3	3.3	4.1	16.9	66.7	85.8	28.8	15.6	25.3	18.1
P56470	Galectin-4 GN=LGALS4	32.3	31.0	16.1	13.9	1.0	0.8	0.5	0.5	1.0	1.9	1.3	2.1	23.7	33.2	42.2	46.7	12.2	19.8	35.9
P24752	Acetyl-CoA acetyltransferase, mitochondrial GN=ACAT1	30.4	47.0	20.4	22.3	1.0	1.0	0.5	0.6	1.0	1.1	0.9	1.5	10.4	14.0	12.2	11.1	22.8	17.0	45.2
P23946	Chymase GN=CMA1	30.3	16.6	11.7	16.2	1.0	0.6	0.4	0.3	1.0	1.5	1.7	1.5	6.5	27.7	31.0	16.5	34.6	16.1	27.3
O60844	Zymogen granule membrane protein 16 GN=ZG16	28.0	0.0	10.8	10.8	1.0	1.0	0.5	0.4	1.0	1.2	1.2	1.7	12.1	77.5	30.3	ND	ND	ND	18.1
Q86TX2	Acyl-coenzyme A thioesterase 1 GN=ACOT1	27.7	37.8	9.5	11.6	1.0	1.0	0.6	0.5	1.0	1.1	1.0	1.3	6.8	18.3	12.8	15.6	11.1	9.6	46.2
P04745	Alpha-amylase 1 GN=AMY1A	23.0	ND	14.1	ND	1.0	1.2	0.7	0.6	ND	ND	ND	ND	13.9	24.4	20.0	ND	ND	ND	10.8
P12724	Eosinophil cationic protein GN=RNASE3	20.9	22.3	28.8	15.0	1.0	1.0	0.6	0.6	1.0	1.1	0.9	1.1	20.9	18.3	47.4	15.3	27.0	20.7	18.4
Q16836	Hydroxyacyl-coenzyme A dehydrogenase, mitochondrial GN=HADH	20.7	9.5	9.9	5.7	1.0	1.1	0.5	0.5	1.0	1.1	1.3	1.5	9.5	22.7	11.1	14.5	2.3	28.7	34.3
O75356	Ectonucleoside triphosphate diphosphohydrolase 5 GN=ENTPD5	20.5	3.3	6.3	2.6	1.0	1.1	0.4	0.4	1.0	1.5	1.0	1.8	3.6	34.9	25.3	ND	ND	ND	47.5
P06865	Beta-hexosaminidase subunit alpha GN=HEXA	19.3	13.4	4.0	7.0	1.0	1.0	0.6	0.6	1.0	1.1	1.8	1.4	17.8	9.7	17.2	3.8	28.1	6.4	60.7
Q9Y6U3	Adseverin GN=SCIN	17.5	7.1	8.8	3.8	1.0	1.1	0.7	0.5	1.0	1.1	1.1	1.4	7.4	15.4	20.7	13.6	4.0	10.2	80.4
Q14508	WAP four-disulfide core domain protein 2 GN=WFDC2	16.5	ND	25.8	ND	1.0	1.2	0.3	0.3	ND	ND	ND	ND	21.8	31.3	37.3	ND	ND	ND	10.8
Q13576	Ras GTPase-activating-like protein IQGAP2 GN=IQGAP2	16.2	18.5	2.0	4.7	1.0	1.1	0.6	0.6	1.0	1.0	1.0	1.4	21.0	17.0	33.3	33.7	10.2	5.5	180.5
Q14002	Carcinoembryonic antigen-related cell adhesion molecule 7 GN=CEACAM7	16.1	1.9	6.8	3.8	1.0	0.6	0.2	0.3	1.0	1.3	1.2	2.0	1.9	39.1	15.1	ND	ND	ND	29.4
P18859	ATP synthase-coupling factor 6, mitochondrial GN=ATP5J	16.0	27.9	30.6	30.6	1.0	1.1	0.6	0.6	1.0	1.2	1.1	1.7	4.8	22.4	4.3	10.3	9.6	19.1	12.6
O95154	Aflatoxin B1 aldehyde reductase member 3 GN=AKR7A3	15.3	2.1	6.3	3.6	1.0	1.3	0.6	0.4	1.0	0.8	0.9	1.1	3.6	7.0	8.6	ND	ND	ND	37.2
P45954	Short/branched chain specific acyl-CoA dehydrogenase, mitochondrial GN=ACADSB	15.3	ND	6.0	ND	1.0	0.8	0.5	0.5	ND	ND	ND	ND	11.6	6.7	25.1	ND	ND	ND	10.8
P50225	Sulfotransferase 1A1 GN=SULT1A1	15.1	ND	13.2	ND	1.0	1.7	0.6	0.6	ND	ND	ND	ND	ND	ND	ND	ND	ND	ND	10.8
Q9HCB6	Spondin-1 GN=SPON1	15.1	15.6	4.6	5.5	1.0	1.3	0.5	0.5	1.0	0.9	2.1	1.2	4.4	39.5	15.6	8.5	46.2	18.1	90.9
P09471	Guanine nucleotide-binding protein G(o) subunit alpha GN=GNAO1	14.2	22.0	7.9	14.7	1.0	1.0	0.4	0.5	1.0	1.5	1.0	0.9	0.4	36.0	33.1	3.0	14.4	25.2	40.0
Q6UWP2	Dehydrogenase/reductase SDR family member 11 GN=DHRS11	13.4	14.3	9.6	5.0	1.0	1.0	0.4	0.5	1.0	1.1	1.0	1.7	5.8	16.2	12.8	9.3	1.9	3.4	28.3
P04066	Tissue alpha-L-fucosidase GN=FUCA1	12.1	6.5	6.4	2.4	1.0	1.2	0.6	0.6	1.0	1.3	1.7	2.1	2.2	18.2	12.2	29.4	21.6	29.2	53.7
O00339	Matrilin-2 GN=MATN2	11.5	5.5	3.1	1.7	1.0	1.2	0.5	0.6	1.0	1.3	1.4	1.8	7.3	0.7	1.4	ND	ND	ND	106.8
P46108	Adapter molecule crk GN=CRK	10.2	20.4	9.5	5.9	1.0	0.9	0.6	0.5	1.0	1.0	0.9	1.1	6.4	0.3	10.3	4.5	13.7	9.3	33.8
P07477	Trypsin-1 GN=PRSS1	9.4	4.3	7.3	7.3	1.0	1.8	0.6	0.6	1.0	1.0	1.4	1.5	14.3	19.3	23.6	0.0	48.5	8.3	26.5
P10301	Ras-related protein R-Ras GN=RRAS	9.3	25.6	12.8	12.8	1.0	1.0	0.5	0.5	1.0	0.9	0.6	1.0	4.0	4.2	3.3	8.9	25.1	23.2	23.5
Q9HAT2	Sialate O-acetylesterase GN=SIAE	8.9	10.4	3.4	6.3	1.0	0.9	0.4	0.5	1.0	1.2	1.3	1.6	4.3	21.4	36.3	4.8	8.6	10.9	58.3
Q96DG6	Carboxymethylenebutenolidase homolog GN=CMBL	8.9	7.8	8.2	11.0	1.0	1.2	0.6	0.5	1.0	0.8	0.6	0.9	5.4	15.8	37.5	24.6	38.7	46.5	28.0
Q96CN7	Isochorismatase domain-containing protein 1 GN=ISOC1	8.6	7.8	5.0	5.0	1.0	1.1	0.4	0.6	1.0	1.1	1.4	1.4	3.2	13.8	9.7	3.8	21.8	6.6	32.2
Q13683	Integrin alpha-7 GN=ITGA7	8.3	7.4	1.0	1.7	1.0	1.0	0.6	0.5	1.0	1.2	1.1	1.0	0.5	13.4	2.9	0.6	10.5	9.8	128.9
O43181	NADH dehydrogenase [ubiquinone] iron-sulfur protein 4, mitochondrial GN=NDUFS4	8.1	4.5	13.7	8.6	1.0	0.9	0.4	0.6	1.0	1.1	1.2	1.8	1.3	42.7	1.5	ND	ND	ND	20.1
P16219	Short-chain specific acyl-CoA dehydrogenase, mitochondrial GN=ACADS	7.8	17.8	6.1	3.2	1.0	1.1	0.5	0.6	1.0	1.6	1.9	2.1	5.9	19.9	18.2	29.8	29.0	26.2	44.3
P10915	Hyaluronan and proteoglycan link protein 1 GN=HAPLN1	7.5	ND	3.7	ND	1.0	0.8	0.3	0.3	ND	ND	ND	ND	3.6	5.5	6.6	ND	ND	ND	10.8
Q7Z7G0	Target of Nesh-SH3 GN=ABI3BP	7.0	7.6	3.7	2.5	1.0	1.0	0.6	0.6	1.0	1.9	4.5	1.9	3.1	12.7	1.1	39.3	41.8	12.5	118.6
O76038	Secretagogin GN=SCGN	6.7	ND	6.5	ND	1.0	2.0	0.7	0.5	ND	ND	ND	ND	11.9	0.0	12.9	ND	ND	ND	10.8
P09601	Heme oxygenase 1 GN=HMOX1	5.5	ND	4.9	ND	1.0	1.2	0.5	0.6	ND	ND	ND	ND	1.7	5.7	4.8	ND	ND	ND	49.2
Q9NSC7	Alpha-N-acetylgalactosaminide alpha-2,6-sialyltransferase 1 GN=ST6GALNAC1	5.3	ND	2.3	ND	1.0	0.9	0.4	0.5	ND	ND	ND	ND	12.1	55.7	38.5	ND	ND	ND	24.9
Q9HCN8	Stromal cell-derived factor 2-like protein 1 GN=SDF2L1	5.0	4.8	8.1	8.1	1.0	1.4	0.5	0.6	1.0	1.0	1.6	1.5	11.2	4.1	9.9	3.5	17.2	20.9	23.6
Q9UPN4	Centrosomal protein of 131 kDa GN=CEP131	4.9	2.4	0.8	0.8	1.0	1.3	0.5	0.6	1.0	0.9	0.3	0.4	2.0	1.8	2.9	ND	ND	ND	122.1
P01275	Glucagon GN=GCG	4.1	ND	7.8	ND	1.0	2.6	0.3	0.3	ND	ND	ND	ND	ND	ND	ND	ND	ND	ND	10.8
P18827	Syndecan-1 GN=SDC1	3.6	ND	5.5	ND	1.0	1.0	0.6	0.6	ND	ND	ND	ND	ND	ND	ND	ND	ND	ND	49.2
P20933	N(4)-(beta-N-acetylglucosaminyl)-L-asparaginase GN=AGA	3.6	8.6	3.2	9.3	1.0	0.9	0.6	0.6	1.0	1.1	1.2	1.4	ND	ND	ND	5.0	6.4	18.0	37.2
O43704	Sulfotransferase family cytosolic 1B member 1 GN=SULT1B1	3.5	4.0	6.1	3.0	1.0	1.0	0.4	0.6	1.0	1.8	1.9	1.8	ND	ND	ND	3.5	4.2	8.1	34.9
P32004	Neural cell adhesion molecule L1 GN=L1CAM	2.9	ND	0.8	ND	1.0	1.1	0.6	0.6	ND	ND	ND	ND	ND	ND	ND	ND	ND	ND	10.8
P10645	Chromogranin-A GN=CHGA	2.8	ND	2.8	ND	1.0	1.6	0.6	0.6	ND	ND	ND	ND	ND	ND	ND	ND	ND	ND	49.2
P28289	Tropomodulin-1 GN=TMOD1	2.7	0.0	2.5	2.8	1.0	0.9	0.3	0.4	1.0	0.9	1.2	1.0	ND	ND	ND	ND	ND	ND	40.5
P24043	Laminin subunit alpha-2 GN=LAMA2	2.7	5.1	0.5	0.8	1.0	1.1	0.6	0.6	1.0	1.4	1.5	1.9	ND	ND	ND	25.8	17.5	18.6	343.7
Q13740	CD166 antigen GN=ALCAM	2.7	ND	1.9	ND	1.0	0.9	0.5	0.5	ND	ND	ND	ND	ND	ND	ND	ND	ND	ND	10.8
O43570	Carbonic anhydrase 12 GN=CA12	2.7	ND	5.1	ND	1.0	1.1	0.6	0.7	ND	ND	ND	ND	ND	ND	ND	ND	ND	ND	49.2
Q8NFL0	UDP-GlcNAc:betaGal beta-1,3-N-acetylglucosaminyltransferase 7 GN=B3GNT7	2.3	ND	2.2	ND	1.0	1.1	0.5	0.5	ND	ND	ND	ND	ND	ND	ND	ND	ND	ND	24.9
A0AV96	RNA-binding protein 47 GN=RBM47	2.3	ND	2.0	ND	1.0	1.1	0.6	0.6	ND	ND	ND	ND	ND	ND	ND	ND	ND	ND	78.1
Q6ZMP0	Thrombospondin type-1 domain-containing protein 4 GN=THSD4	2.3	ND	1.1	ND	1.0	0.8	0.5	0.5	ND	ND	ND	ND	ND	ND	ND	ND	ND	ND	10.2
P06870	Kallikrein-1 GN=KLK1	2.2	ND	3.8	ND	1.0	1.2	0.6	0.6	ND	ND	ND	ND	ND	ND	ND	ND	ND	ND	76.7
Q96FZ7	Charged multivesicular body protein 6 GN=CHMP6	2.1	ND	6.5	ND	1.0	1.1	0.6	0.6	ND	ND	ND	ND	ND	ND	ND	ND	ND	ND	110.3
Q96IJ6	Mannose-1-phosphate guanyltransferase alpha GN=GMPPA	2.0	7.9	3.3	5.5	1.0	1.0	0.6	0.6	1.0	0.9	0.9	1.6	ND	ND	ND	12.9	5.0	10.9	46.3
P09417	Dihydropteridine reductase GN=QDPR	1.9	1.8	3.3	3.3	1.0	0.9	0.6	0.6	1.0	0.9	0.7	0.7	ND	ND	ND	ND	ND	ND	25.8
Q5JTB6	Placenta-specific protein 9 GN=PLAC9	1.8	ND	10.3	ND	1.0	0.7	0.5	0.4	ND	ND	ND	ND	ND	ND	ND	ND	ND	ND	10.8
P01860	Ig gamma-3 chain C region GN=IGHG3	481.6	657.2	38.7	38.7	1.0	0.3	0.5	0.3	1.0	1.1	2.8	2.1	48.1	37.5	47.6	29.1	58.7	26.9	41.3
P07602	Prosaposin GN=PSAP	125.2	151.1	18.9	19.9	1.0	0.9	1.0	0.8	1.0	1.1	1.7	1.7	5.8	19.4	19.6	7.0	21.5	13.5	58.1
P07686	Beta-hexosaminidase subunit beta GN=HEXB	53.0	39.6	10.8	9.2	1.0	0.9	0.5	0.8	1.0	1.1	1.7	1.7	3.8	26.1	10.3	6.4	17.6	17.4	63.1
P07305	Histone H1.0 GN=H1F0	24.7	24.0	11.3	11.9	1.0	1.6	0.9	2.6	1.0	0.8	1.6	1.9	27.9	23.8	41.7	10.0	6.3	15.2	20.9
Q9C075	Keratin, type I cytoskeletal 23 GN=KRT23	15.5	13.4	17.8	11.1	1.0	0.9	2.2	1.5	1.0	1.1	1.6	3.3	23.6	59.2	71.1	5.3	16.1	23.9	48.1
P83881	60S ribosomal protein L36a GN=RPL36A	4.1	9.8	16.0	16.0	1.0	0.9	0.8	1.0	1.0	1.0	1.7	1.8	14.3	19.9	3.0	14.3	22.0	4.8	12.4
Q9GIY3	HLA class II histocompatibility antigen, DRB1-14 beta chain GN=HLA-DRB1	6.9	5.3	3.8	10.5	1.0	0.6	0.6	0.6	1.0	1.1	1.6	1.8	41.2	53.8	31.6	ND	ND	ND	30.1
P05062	Fructose-bisphosphate aldolase B GN=ALDOB	ND	3.5	ND	6.6	ND	ND	ND	ND	1.0	1.1	1.8	2.1	ND	ND	ND	15.6	41.7	32.3	39.4
O95758	Polypyrimidine tract-binding protein 3 GN=PTBP3	6.8	3.2	3.3	1.5	1.0	0.8	1.2	0.8	1.0	1.0	1.6	1.6	7.5	23.4	20.9	ND	ND	ND	59.7
P02741	C-reactive protein GN=CRP	ND	3.2	ND	4.5	ND	ND	ND	ND	1.0	1.0	2.3	3.4	ND	ND	ND	ND	ND	ND	25.0
Q9BS40	Latexin GN=LXN	ND	3.0	ND	5.4	ND	ND	ND	ND	1.0	1.4	2.2	2.1	ND	ND	ND	ND	ND	ND	25.7
Q9P2A4	ABI gene family member 3 GN=ABI3	1.8	2.3	2.2	2.2	1.0	1.0	1.0	0.9	1.0	1.1	1.7	1.9	ND	ND	ND	ND	ND	ND	39.0
P62736	Actin, aortic smooth muscle GN=ACTA2	1997.0	4259.7	61.8	61.5	1.0	1.1	0.8	0.8	1.0	1.0	0.6	0.6	12.5	30.9	49.3	11.0	78.0	68.3	42.0
Q01995	Transgelin GN=TAGLN	646.9	1301.4	67.7	68.7	1.0	1.2	0.9	0.6	1.0	1.1	0.6	0.5	13.0	12.2	29.8	4.8	45.4	54.5	22.6
P07951	Tropomyosin beta chain GN=TPM2	765.9	1254.6	44.7	55.6	1.0	1.0	0.7	0.8	1.0	1.0	0.6	0.6	15.0	18.9	27.1	13.9	66.2	36.3	32.8
O15061	Synemin GN=SYNM	19.3	104.3	3.0	11.8	1.0	1.6	0.7	0.7	1.0	0.9	0.6	0.6	14.0	39.2	25.0	22.3	45.1	40.2	172.7
Q15005	Signal peptidase complex subunit 2 GN=SPCS2	12.4	8.3	8.4	8.4	1.0	1.0	0.8	0.8	1.0	0.9	0.4	0.5	12.4	1.0	3.0	4.0	45.5	27.9	25.0

The relative abundance was calculated with TMT-126 labeled sample set as 1.000 in each experiment (ND, not detected)

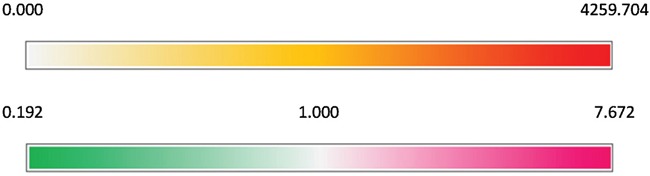

Of the 146 DEPs, the expression levels of at least 66 proteins (e.g. HMG1, CEA, C-reactive protein, etc.) have been previously reported to be associated with occurrence or progression of CRC (Supplementary Table S2), which provides strong support for the reliability of our MS data.

### Over-representation analysis revealed that the expression of extracellular matrix, exosome and contractile fiber proteins are associated with distant recurrence in Stage III CRC

To identify the recurrence-related physiological processes implicated by the DEPs, we next clustered the proteins into GO categories, biological pathways and protein complexes using bioinformatics tools.

First, we examined GO category over-representation of the upregulated, downregulated and overall DEPs using the ConsensusPathDB server (http://consensuspathdb.org/); only GO level 4 categories were screened for precise annotation.

As shown in Table 3, the up- and downregulated proteins in the Stage IIIB group show significantly different over-representation. The samples from Stage IIIB distant recurrence cases overexpressed proteins involved in “defense response to fungus”, “RAGE receptor binding”, “RNA binding” and the “box C/D snoRNP complex”. In contrast, proteins related to “extracellular matrix organization”, “immunoglobulin receptor binding”, “extracellular vesicular exosome” and the “IgM/A complex” were under-expressed.

**Table 3:** GO level 4 categories over-represented in Stage IIIB and IIIC DEPs

Stage	Gene Ontology	Level 4 Categories	Up		Down		Total	
			gene count	*q*-value	gene count	*q*-value	gene count	*q*-value
**IIIB**	**Biological Processes**	defense response to fungus	4 (14.8%)	5.37E-05	NA	NSS	NA	NSS
		extracellular matrix organization	NA	NSS	13 (3.5%)	7.53E-06	14 (3.7%)	4.86E-05
		antibacterial humoral response	NA	NSS	NA	NSS	6 (16.7%)	4.86E-05
		antimicrobial humoral response	NA	NSS	NA	NSS	6 (15.0%)	4.86E-05
	**Molecular Functions**	RAGE receptor binding	4 (36.4%)	1.59E-07	NA	NSS	4 (36.4%)	3.92E-05
		RNA binding	17 (1.1%)	1.65E-07	NA	NSS	NA	NSS
		immunoglobulin receptor binding	NA	NSS	3 (42.9%)	1.24E-04	3 (42.9%)	3.07E-04
	**Cellular Components**	box C/D snoRNP complex	2 (50.0%)	8.69E-04	NA	NSS	NA	NSS
		extracellular vesicular exosome	NA	NSS	47 (1.7%)	4.72E-15	60 (2.2%)	1.99E-15
		IgM immunoglobulin complex	NA	NSS	2 (100.0%)	2.25E-04	2 (100.0%)	6.81E-04
		pentameric IgM immunoglobulin complex	NA	NSS	2 (100.0%)	2.25E-04	2 (100.0%)	6.81E-04
		hexameric IgM immunoglobulin complex	NA	NSS	2 (100.0%)	2.25E-04	2 (100.0%)	6.81E-04
		IgA immunoglobulin complex	NA	NSS	2 (66.7%)	3.85E-04	NA	NSS
		monomeric IgA immunoglobulin complex	NA	NSS	2 (66.7%)	3.85E-04	NA	NSS
		polymeric IgA immunoglobulin complex	NA	NSS	2 (66.7%)	3.85E-04	NA	NSS
**IIIC**	**Biological Processes**	muscle system process	NA	NSS	4 (1.2%)	1.10E-04	NA	NSS
	**Cellular Components**	contractile fiber	NA	NSS	5 (2.3%)	5.92E-08	5 (2.3%)	4.38E-05
		cytoskeleton	NA	NSS	6 (0.3%)	7.14E-05	9 (0.5%)	5.87E-04

The percentages in the parentheses reflect the proportion of the over-represented genes of the input to the total gene number of corresponding GO categories. NA, not available, NSS, not statistically significant.

As mentioned above, only 21 DEPs were identified in the Stage IIIC group. The upregulated proteins showed no significant over-representation among GO level 4 categories. However, downregulated proteins involved in “muscle system process”, “contractile fiber” and “cytoskeleton” were enriched (Table 3).

To get a glimpse of the biological pathways involved in distant recurrence in Stage III CRC, ConsensusPathDB was used to map the DEPs to pathway databases. As shown in Table 4, fatty acid degradation-related and extracellular matrix-related pathways were over-represented among the DEPs in the Stage IIIB group, while muscle contraction-related pathways were enriched in Stage IIIC DEPs.

**Table 4:** Biological pathways over-represented in Stage IIIB and IIIC DEPs

Stage	Pathways	Up		Down		Total	
		gene count	*q*-value	gene count	*q*-value	gene count	*q*-value
**IIIB**	Fatty acid degradation - Homo sapiens (human)	NA	NSS	6 (13.6%)	1.20E-04	NA	NSS
	Metabolism	NA	NSS	25 (1.7%)	4.07E-04	NA	NSS
	Endohydrolysis of 1,4-alpha-D-glucosidic linkages in polysaccharides by alpha-amylase	NA	NSS	3 (50.0%)	5.44E-04	NA	NSS
	Extracellular matrix organization	NA	NSS	10 (3.8%)	6.47E-04	NA	NSS
	Butyrate Metabolism	NA	NSS	3 (37.5%)	6.47E-04	NA	NSS
	Mitochondrial Beta-Oxidation of Short Chain Saturated Fatty Acids	NA	NSS	3 (37.5%)	6.47E-04	NA	NSS
	Short-chain 3-hydroxyacyl-CoA dehydrogenase deficiency (SCHAD)	NA	NSS	3 (37.5%)	6.47E-04	NA	NSS
	Saturated fatty acids beta-oxidation	NA	NSS	4 (16.0%)	7.51E-04	NA	NSS
	Digestion of dietary carbohydrate	NA	NSS	3 (33.3%)	7.51E-04	NA	NSS
**IIIC**	Muscle contraction	NA	NSS	4 (7.7%)	1.42E-07	4 (7.7%)	1.88E-05
	Smooth Muscle Contraction	NA	NSS	3 (12.5%)	1.41E-06	3 (12.5%)	6.35E-05
	Striated Muscle Contraction	NA	NSS	3 (7.9%)	3.91E-06	3 (7.9%)	1.74E-04

The percentages in the parentheses reflect the proportion of the over-represented genes of the input to the total gene number of corresponding pathways. NA, not available, NSS, not statistically significant.

To analyze the potential cooperation between the DEPs at a molecular level, we finally mapped the DEPs to protein complex databases, and identified that the Stage IIIB DEPs over-represented several protein complexes (Table 5) involved in ribosome biogenesis (Nop56p complex), chromatin metabolism (HMGB1 and CDCA5 complexes), alcohol metabolism (alcohol dehydrogenase) and extracellular matrix (laminin complexes), while no significant protein complex over-representation was observed in the Stage IIIC DEPs.

**Table 5:** Protein complexes over-represented in Stage IIIB DEPs

Complexes	Up		Down		Total	
	gene count	*q*-value	gene count	*q*-value	gene count	*q*-value
Nop56p-associated pre-rRNA complex	5 (4.7%)	4.12E-04	NA	NSS	NA	NSS
HMGB1-HMGB2-HSC70-ERP60-GAPDH complex	2 (40.0%)	7.83E-04	NA	NSS	NA	NSS
CDCA5-PDS5A-RAD21-SMC1A-PDS5B-SMC3 complex	2 (33.3%)	7.83E-04	NA	NSS	NA	NSS
L1:ALCAM	NA	NSS	2 (100.0%)	4.44E-04	NA	NSS
alcohol dehydrogenase 1 (class I), alpha/beta dimer	NA	NSS	2 (66.7%)	4.44E-04	NA	NSS
Laminin-221	NA	NSS	2 (66.7%)	4.44E-04	NA	NSS
alpha7×1/beta1 Integrin/Laminin 2	NA	NSS	2 (40.0%)	5.51E-04	NA	NSS
alpha7×1/beta1 Integrin/Laminin 11	NA	NSS	2 (40.0%)	5.51E-04	NA	NSS
alpha6/beta1 Integrin/Laminin 4	NA	NSS	2 (40.0%)	5.51E-04	NA	NSS
alpha6/beta4 Integrin/Laminin 4	NA	NSS	2 (40.0%)	5.51E-04	NA	NSS
alpha3/beta1 Integrin/Laminin 4	NA	NSS	2 (40.0%)	5.51E-04	NA	NSS

The percentages in the parentheses reflect the proportion of the over-represented genes of the input to the total gene number of corresponding protein complexes. NA, not available, NSS, not statistically significant.

### Interaction network construction revealed hub proteins potentially regulating or cooperating with the DEPs

**Figure 1:**
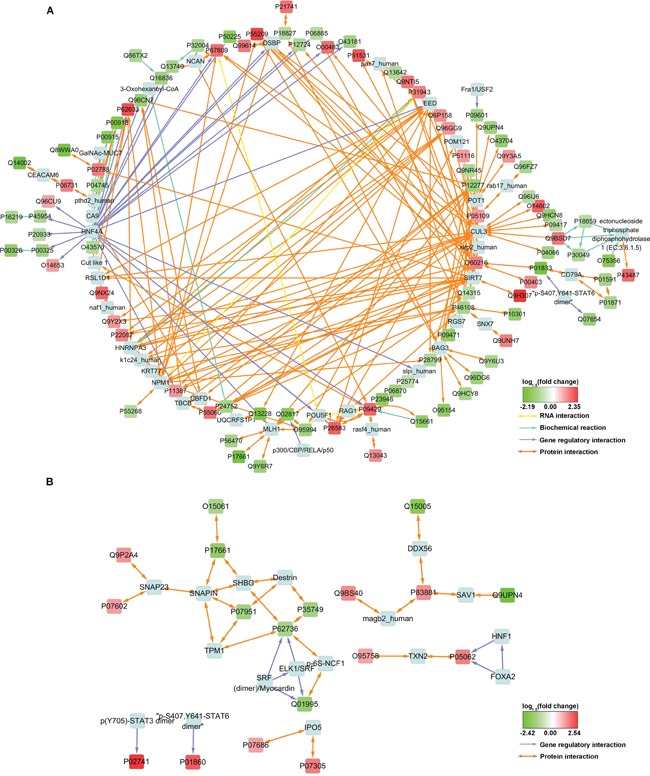
Interaction network constructed with some of the MS-identified DEPs Input nodes were colored with log1.5 (fold change) and designated with UniProt Accessions. Intermediate nodes of exogenous proteins introduced by Consensus PathDB were light blue and designated with conventional names. For protein expression measurement information, see Supplementary Table S4.

To reveal the potential interactions between the DEPs, interaction networks were constructed (Figure 1). The generated networks not only contain the distant recurrence-associated DEPs (“input nodes”), but also some highly correlative interactors or transcription factors (“intermediate nodes”) that were not identified or whose expression levels were unaltered in MS. In the network of the Stage IIIB DEPs (Figure 1A), the proteins EED, CUL3, SIRT7, BAG3, POT1 and P55209 (NAP1L1) serve as hubs that converge the majority of represented protein interactions. Additionally, the transcription factor HNF4A potentially regulates as many as 18 DEPs, most of which were downregulated in patients who developed distant recurrence.

The induced network for Stage IIIC DEPs is less complicated and no apparent hub nodes were observed (Figure 1B). However, this network still revealed some potential interactors, such as SNAP23, SHBG, Destrin and TXN2 etc., and two SRF complexes that may potentially be involved in the transcriptional regulation of P62736 (ACTA2) and Q01995 (Transgelin).

### IHC and statistical analysis revealed that R-Ras and Transgelin expression correlate positively with post-surgical prognosis in Stage III CRC

In the 146 DEPs, 107 proteins were both detected in IIIB and IIIC groups. Using the relative abundance values of the 107 proteins from the two MS experiments, *t*-tests were performed to find out the proteins showing statistically differential expression in distant recurrence patients regardless of CRC subdivision. As shown in Table 6, 18 proteins were identified. In these proteins, we are interested in R-Ras and Transgelin, and their existence is supported by their unique peptide MS/MS spectra (examples are shown in Supplementary Figure S2). As distant recurrence is associated with poor survival rates, we considered the possibility that the protein level of R-Ras or Transgelin might serve as post-surgical prognostic biomarkers in Stage III CRC.

**Table 6:** Proteins which show statistically differential expression between good outcome and distant recurrence of Stage IIIB and IIIC CRC patients

Accession	Description	*p*-value	Relative Abundance	
			Mean of G1-G4	Mean of P1-P4
P17661	Desmin GN=DES	5.51E-05	0.991	0.412
Q14315	Filamin-C GN=FLNC	7.37E-05	0.966	0.657
P09417	Dihydropteridine reductase GN=QDPR	2.16E-04	0.955	0.650
P35749	Myosin-11 GN=MYH11	5.80E-04	1.027	0.548
P07951	Tropomyosin beta chain GN=TPM2	6.90E-04	1.006	0.668
P24844	Myosin regulatory light polypeptide 9 GN=MYL9	7.72E-04	1.052	0.672
P62736	Actin, aortic smooth muscle GN=ACTA2	9.25E-04	1.012	0.679
Q9UPN4	Centrosomal protein of 131 kDa GN=CEP131	1.98E-03	1.071	0.478
Q01995	Transgelin GN=TAGLN	8.53E-03	1.094	0.664
Q15005	Signal peptidase complex subunit 2 GN=SPCS2	1.49E-02	0.972	0.624
Q9UNH7	Sorting nexin-6 GN=SNX6	1.85E-02	0.969	1.687
O15061	Synemin GN=SYNM	1.86E-02	1.136	0.636
P10301	Ras-related protein R-Ras GN=RRAS	2.50E-02	0.994	0.626
Q96DG6	Carboxymethylenebutenolidase homolog GN=CMBL	2.79E-02	1.016	0.657
Q9C075	Keratin, type I cytoskeletal 23 GN=KRT23	3.28E-02	0.981	2.145
Q9HCY8	Protein S100-A14 GN=S100A14	3.96E-02	0.918	0.612
Q6WKZ4	Rab11 family-interacting protein 1 GN=RAB11FIP1	4.01E-02	0.979	1.636
P11387	DNA topoisomerase 1 GN=TOP1	4.91E-02	0.918	1.339

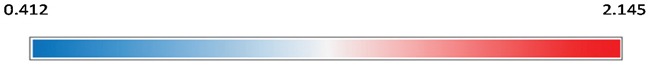

To test the idea, tumor and para-tumor tissues from 192 eligible Stage III CRC patients were analyzed. The patients were dichotomized as high or low protein expression based on IHC staining (Supplementary Figures S3 and S4). We observed that low expression of R-Ras or Transgelin was correlated with the tumor tissues, but not with the para-tumor tissues (Supplementary Table S3).

We next assessed the association of R-Ras or Transgelin expression with CRC patients' clinicopathological features. Unpaired *t*-tests showed that their expression is not associated with factors reflecting the general condition of the patients, such as gender and age, neither with the tumor location or differentiation degree (Tables 7 and 8). However, the levels of R-Ras and Transgelin were associated with the plasma CEA level.

**Table 7:** Relationship between R-Ras expression and clinicopathological features of Stage III CRC

Variables	Number (n)	R-Ras expression		*p*-value
		Positive (n=83)	Negative (n=109)	
**Gender**				0.389
**Male**	112	45	67	
**Female**	80	38	42	
**Ages (years)**				0.528
**>=65**	91	42	49	
**<65**	101	41	60	
**Tumor location**				0.661
**ascending colon**	97	41	56	
**transverse colon**	7	2	5	
**descending colon**	88	40	48	
**CEA level (mg/L)**		7.20±7.00	17.46±34.66	0.008^*^
**Tumor differentiation**				0.886
**Well**	21	10	11	
**Moderate**	135	57	78	
**Poor**	36	16	20	
**AJCC stage**				0.751
**IIIA**	7	4	3	
**IIIB**	124	53	71	
**IIIC**	61	26	35	

* Statistically significant (*p*<0.05)

**Table 8:** Relationship between Transgelin expression and clinicopathological features of Stage III CRC

Variables	Number (n)	Transgelin expression		*p*-value
		Positive (n=84)	Negative (n=108)	
**Gender**				0.461
**Male**	112	52	60	
**Female**	80	32	48	
**Ages (years)**				0.841
**>=65**	91	41	50	
**<65**	101	43	58	
**Tumor location**				0.767
**ascending colon**	97	42	55	
**transverse colon**	7	4	3	
**descending colon**	88	38	50	
**CEA level (mg/L)**		7.11±7.45	22.11±32.87	<0.001^*^
**Tumor differentiation**				0.285
**Well**	21	7	14	
**Moderate**	135	64	71	
**Poor**	36	13	23	
**AJCC stage**				0.027^*^
**IIIA**	7	5	2	
**IIIB**	124	60	64	
**IIIC**	61	19	42	

* Statistically significant (p<0.05)

**Figure 2:**
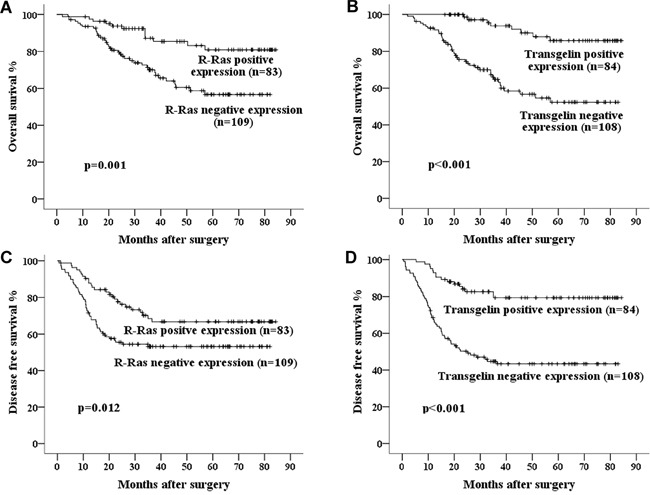
Kaplan–Meier curves of 5-year OS and DFS in patients with **A, C.** R-Ras negative and positive expression; **B, D.** Transgelin negative and positive expression. Poorer survival was seen in the patients whose tumors showed negative expression of R-Ras or Transgelin.

To evaluate the correlation of R-Ras or Transgelin with patients' survival, Kaplan-Meier analysis was performed and we observed that low R-Ras or Transgelin levels were positively correlated with survival of patients with Stage III CRC (Figure 2). To identify whether R-Ras or Transgelin expression serves as an independent predictor of patients' survival, univariate and multivariate analysis were conducted. As shown in Tables 9 and 10, univariate analysis showed that the expression of R-Ras and Transgelin, CEA level, tumor differentiation and AJCC stage were significant prognostic factors for OS and DFS in patients undergoing “curative” surgery. However, in multivariate Cox-regression analyses, only R-Ras and AJCC stage were prognostic factors for OS, while only Transgelin and tumor differentiation were prognostic factors for DFS.

**Table 9:** Univariate and multivariate analyses of individual parameters for correlations with OS rate

Variables	Univariate			Multivariate		
	HR	CI (95%)	*p*-value	HR	CI (95%)	*p*-value
**Gender**	0.88	0.50-1.57	0.668			
**Age**	0.90	0.51-1.58	0.712			
**Tumor location**	0.78	0.58-1.04	0.093			
**R-Ras expression**	2.86	1.49-5.50	0.001^*^	2.07	1.04-4.10	0.037^*^
**Transgelin expression**	3.04	1.56-5.77	0.001^*^	1.95	0.98-3.87	0.059
**CEA level**	1.77	1.02-3.17	0.044^*^	1.20	0.66-2.19	0.557
**Tumor differentiation**	2.08	1.07-4.04	0.028^*^	2.06	0.95-4.49	0.069
**AJCC stage**	2.18	1.28-3.73	0.004^*^	2.02	1.14-3.57	0.016^*^

Cox proportional hazards model. HR: hazard ratio; CI: confidence interval

* Statistically significant (*p*<0.05).

**Table 10:** Univariate and multivariate analyses of individual parameters for correlations with DFS rate

Variables	Univariate			Multivariate		
	HR	CI (95%)	*p*-value	HR	CI (95%)	*p*-value
**Gender**	0.73	0.46-1.18	0.196			
**Age**	1.12	0.70-1.73	0.687			
**Tumor location**	0.82	0.65-1.04	0.108			
**R-Ras expression**	1.92	1.18-3.12	0.008^*^	1.40	0.83-2.36	0.211
**Transgelin expression**	2.43	1.44-3.97	0.001^*^	1.85	1.07-3.20	0.028^*^
**CEA level**	1.78	1.12-2.83	0.014^*^	1.39	0.86-2.26	0.179
**Tumor differentiation**	1.65	1.05-2.59	0.032^*^	1.75	1.04-2.95	0.034^*^
**AJCC stage**	1.72	1.11-2.66	0.014^*^	1.57	0.98-2.51	0.061

Cox proportional hazards model. HR: hazard ratio; CI: confidence interval

* Statistically significant (*p*<0.05).

**Figure 3:**
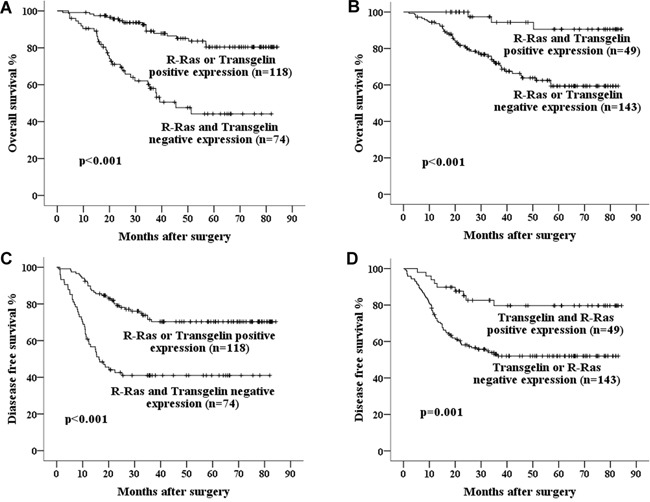
Kaplan–Meier curves of 5-year OS in patients with **A.** Either R-Ras or Transgelin positive expression vs. both R-Ras and Transgelin negative expression; **B.** Both R-Ras and Transgelin positive expression vs. either R-Ras or Transgelin negative expression. And curves of 5- year DFS in patients with **C.** Either R-Ras or Transgelin positive expression vs. both R-Ras and Transgelin negative expression; **D.** Both R-Ras and Transgelin positive expression vs. either R-Ras or Transgelin negative expression. Concurrent positive expression of R-Ras and Transgelin is associated with much better prognosis.

Finally, we assessed the combined prognostic value of R-Ras and Transgelin for survival in Stage III CRC. Kaplan-Meier Method analysis revealed that concurrent downregulation of R-Ras and Transgelin was correlated with significantly lower 5-year OS and DFS, while concurrent positive expression was associated with a better prognosis (Figure 2 and 3).

### R-Ras promotes migration and invasion in CRC cell lines

In the proteomic and statistical studies described above, we found that under-expression of R-Ras protein was associated with distant recurrence and poor prognosis in Stage III CRC. Therefore, we investigated the mechanism by which the R-Ras protein may be involved in the development of cancer.

**Figure 4:**
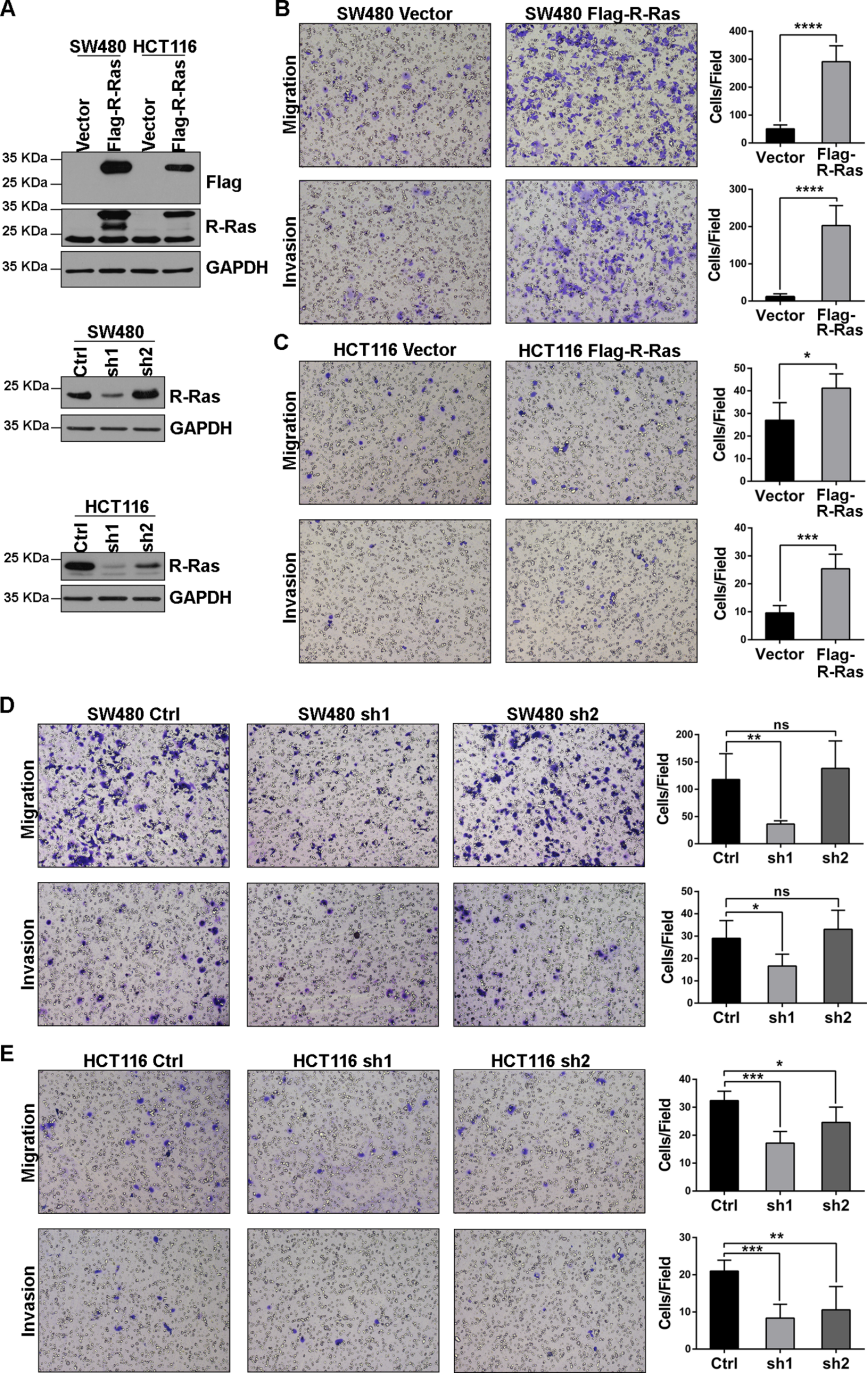
R-Ras promotes the migration and invasion of CRC cell lines **A.** Western blots showed stable over-expression of 3×Flag-R-Ras and shRNA down-regulated endogenous R-Ras; sh2 did not significantly affect endogenous R-Ras in SW480 cells. **B, C.** Over-expression of R-Ras enhanced the migration and invasion of SW480 and HCT116 cells in Transwell assays. **D, E.** Knockdown of R-Ras attenuated the migration and invasion of SW480 and HCT116 cells in Transwell assays. Significance was evaluated using the Student's *t*-test. *, *p* < 0.05, **, *p* < 0.01, ***, *p* < 0.001, ****, *p* < 0.0001, ns, not significant.

We constructed stable R-Ras knockdown cell lines using lentivirus-mediated RNAi. In both SW480 and HCT116 cells, when we stably expressed 3×Flag-R-Ras in the cell lines at a level comparable with the endogenous (Figure 4A), enhanced migration and invasion were observed in the Transwell assays (Figure 4B, 4C). Consistent with this finding, when endogenous R-Ras was down-regulated using shRNAs, the migration and invasion potential of the cell lines were significantly attenuated (Figure 4D, 4E). Additionally, the CCK8 assay revealed that neither knockdown nor over-expression of R-Ras altered the proliferation of SW480 or HCT116 cells (Supplementary Figure S5). These results suggest R-Ras does not participate as either a causal or critical factor in distant recurrence and its downregulation occurs in parallel with or as a result of the acquisition of enhanced metastatic ability.

## DISCUSSION

### Differences in distant recurrence-associated DEPs between Stage IIIB and IIIC implies molecular transformation during CRC development

Several previous proteomics studies have been carried out using specimens from patients with CRC (reviewed in [7]), however, none have examined the molecular differences in tumor tissues from patients with Stage III who achieved a good outcome and those who suffered distant recurrence, or assessed the differences separately in IIIB and IIIC subgroups. In this study, we used TMT-based MS to investigate distant recurrence-associated DEPs in patients with Stage IIIB and IIIC CRC. We identified a much larger repertoire of DEPs in Stage IIIB than in Stage IIIC CRC, with an overlap of only four proteins.

The subdivisions of Stage IIIA, IIIB and IIIC were introduced in the 6^th^ edition of the TNM staging system [8]. The number of positive lymph nodes distinguishes Stage IIIB and IIIC, and this numerical cutoff was determined on the basis of 5-year survival rates [5, 6]. In the 7^th^ edition of the TNM, T4bN1 was classified as IIIC [9]. This reclassification was not involved in the MS experiments of this study.

The presence of cancer cells in the regional lymph nodes is a consequence of tumor-host interactions [10]. In this study, 129 post-surgical distant recurrence-associated DEPs were identified in patients with Stage IIIB CRC. However, only 21 DEPs were identified in patients with Stage IIIC. Three proteins were downregulated in patients with distant recurrence in both the Stage IIIB and IIIC groups: MYH11, DES and CEP131. MYH11 and DES, together with the Stage IIIC-specific downregulated proteins ACTA2, TPM2 and SYNM, are involved in muscle contraction and are reported be intensively expressed in pericytes that surround carcinomatous glands and microvessels [11]. Downregulation of these proteins and Transgelin, which was validated by IHC, suggests that pericyte recruitment defect, which leads to leaky microvessel walls and promotes tumor metastasis [12]. In patients with Stage IIIB CRC who suffered distant recurrence, the levels of ACTA2, TPM2, SYNM and Transgelin were also lower than those of patients with a good outcome, though these differences did not exceed the 1.5-fold change threshold. This evidence indicates that in the later stages of CRC development characterized by more extensive regional lymph node invasion (e.g. Stage IIIC), weakening host defenses around vessels plays a dominant role in determining distant recurrence.

GO analysis also revealed 14 proteins involved in extracellular matrix organization that were differentially expressed in patients with Stage IIIB who suffered distant recurrence. Most of these proteins, except cartilage-specific ACAN, were downregulated, which is consistent with previous reports [13]. The remaining 13 proteins, except for MYH11, were either undetectable or unaltered in patients with Stage IIIC who suffered distant recurrence.

The GO Cellular Components Category “extracellular vesicular exosome” was over-represented among Stage IIIB distant recurrence-associated downregulated proteins, as well as among total DEPs. These 60 exosomal proteins accounted for almost half of the total Stage IIIB DEPs, and most of these (47 out of 60) were under-expressed. It has been reported the exosome level in the blood of patients with CRC correlates negatively with prognosis [14]. Together with our discovery, this data indicates that primary tumors prone to metastasis may possess the propensity to release large quantities of exosomes. According to previous reports [15], exosome release facilitates cellular communication and horizontal gene transfer, and therefore modulates the tumor microenvironment and promotes malignancy. On the other hand, pathway over-representation analysis revealed that proteins involved in “fatty acid degradation” were also enriched in patients with Stage IIIB who developed distant recurrence. Cancer cells require an extra supply of fatty acids for rapid proliferation and other activities [16], such as exosome secretion – as identified in this study. Downregulation of fatty acid degradation proteins would be one way of increasing the supply of fatty acids. Notably, the alternative mechanism, increased expression of fatty acid synthetases (e.g. ACLY and FASN etc.) was not observed in this study.

Proteins of the IgM and IgA complexes (IGHA2, IGHM and IGJ) were also downregulated in patients with Stage IIIB who suffered distant recurrence, but not in the Stage IIIC group. Since IgM and IgA are secreted from plasma cells to intestinal mucous membrane surfaces, decreased levels of IgM and IgA may reflect severe mucosal dysfunction in patients with Stage IIIB who suffer distant recurrence.

All of the evidence discussed above indicates that in the earlier stages of CRC development characterized by limited regional lymph node invasion (e.g. Stage IIIB), large biochemical distortions in cancer cells themselves, involving the extracellular matrix, exosomes and fatty acid mechanism, confer metastatic potential to CRC.

### Transcriptional regulation mediated by HNF4A may play a pivotal role in triggering distant recurrence in Stage IIIB CRC

HNF4A is a transcription factor potentially required for the development of pancreas and liver [17]. Mutations in this protein have been associated with diseases, such as MODY1 [18], NIDDM [19] and FRTS4 [20]. In 2012, a focal amplification enrichment was identified near *HNF4A* gene in CRC tissues [21], indicating HNF4A may function in CRC. Further in 2015, Tian et al. reported that the HNF4A promoter is aberrantly hypermethylated in CRC [22].

In this study, we only detected HNF4A protein in IIIB samples and induced interaction network revealed there are 18 Stage IIIB DEPs potentially under the control of HNF4A, most of which were under-expressed. However, quantification results did not identify HNF4A as a DEP. This indicates that other factors, such as chromatin recruitment or post-translational modification, were probably involved in the regulation of HNF4A's function.

### R-Ras and Transgelin participate in distant recurrence via different mechanisms

R-Ras is a member of the Ras GTPase family, but is less well-characterized than K-, H- and N-Ras [23]; the function of R-Ras in CRC has not yet been determined. The role of Transgelin in cancer is controversial. Some researchers consider Transgelin to be a tumor suppressor [24], while others have reported it promotes cancer cell migration and invasion [25, 26]. In this study, we showed the expression of R-Ras and Transgelin positively correlated with the survival of patients with Stage III CRC.

As illustrated in Supplementary Figures S3 and S4, R-Ras and Transgelin showed different expression patterns in the para-tumor tissues. R-Ras was mainly expressed in crypt epithelial cells, which are the main origin of CRC (e.g. adenocarcinoma, > 90% of CRC cases [27]), while Transgelin was mainly expressed in the cells of the lamina propria. In tumor tissues, positive R-Ras signal was mainly detected in proliferating cancer cells, while Transgelin staining was concentrated in the “grids” that separate adenocarcinomatous glands.

As our results showed a low level of R-Ras was associated with distant recurrence in Stage III CRC, we initially supposed that R-Ras functions as a tumor suppressor. However, the Transwell assays revealed that R-Ras actually promoted the migration and invasion of CRC cell lines (Figure 4). One explanation for this paradox is that down-regulation of R-Ras accompanies the acquisition of increased metastatic ability and does not determine the development of cancer by itself. One other possibility, that the Transwell assay does not reproduce the *in vivo* behavior of Stage III CRC cells, also exists.

Transgelin is not highly expressed in CRC cells; instead, it was detected in the cells of the lamina propria. Transgelin may play an important role in maintaining an intact barrier around the primary site formed by cancerous crypt epithelial cells, which may prevent the metastasis of CRC.

## MATERIALS AND METHODS

### Reagents

Sequencing-grade trypsin and endoproteinase Lys-C were purchased from Roche (Penzberg, Upper Bavaria, Germany) and Promega (Fitchburg, WI, USA), respectively. TMT Mass Tagging Kits and Reagents were purchased from Thermo Scientific Pierce (Rockford, IL, USA). Crystal violet, Transwell permeable supports (24-well) and Cell Counting Kit −8 (CCK-8) were purchased from Sigma-Aldrich (St. Louis, MO, USA). Matrigel was purchased from BD Biosciences (Franklin Lakes, NJ, USA). Puromycin was purchased from Invitrogen (Middlesex, MA, USA).

Antibodies used in this research were: anti-FLAG (F1804, Sigma-Aldrich); anti-Transgelin (ab14106; Abcam, Cambridge, Cambridgeshire, UK); anti-R-Ras (sc-523; Santa Cruz Biotechnology, Dallas, TX, USA) and anti-R-Ras (#8446; Cell Signaling Technology, Danvers, MA, USA).

Acclaim PepMap RSLC columns were purchased from Thermo Scientific Dionex (Sunnyvale, CA, USA). Reversed-phase column Oasis® HLB and Xbridge BEH300 C18 columns were purchased from Waters (Milford, MA, USA).

### Cell lines

HEK293T, SW480 and HCT116 cells were purchased from the China Infrastructure of Cell Line Resources (Chinese Academy of Medical Sciences, Beijing, China). HEK293T and SW480 cells were cultured in Dulbecco's Modified Eagle Medium supplemented with 10% FBS. HCT116 was cultured in Iscove's Modified Dulbecco's Medium supplemented with 10% FBS.

### Lentivirus-mediated knockdown and over-expression

Three plasmids bearing shRNAs targeting different sections of R-Ras mRNA (NM_006270.4) were constructed using the lentivirus-derived vector pLv-shRNA-KP (Era Biotech, Shanghai, China). Targeting sequences were CCACTATTGAGGACTCCTACA and CCTGCTGGTGTTCGCCATTAA. The control sequence was CAACAAGATGAAGAGCACCAA.

To construct the R-Ras over-expressing plasmid, the ORF of *R-Ras* (NM_006270.4) was cloned into the lentivirus-derived vector pLv-CP06 (Era Biotech, Shanghai, China) to express exogenous R-Ras with an N-terminal 3× Flag tag.

Lentivirus particles were produced by co-transfection of lentivirus vector and the packaging plasmids pCMV-VSV-G, pCMV-Gag-Pol and pRSV-Rev (Era Biotech, Shanghai, China) into HEK293T cells. Viral supernatant was harvested at 48 h and 72 h post-transfection. Cells was transduced with the supernatant and 8 ug/ml polybrene. At 48 h post-transduction, stable SW480 and HCT116 cells were selected by 10 μg/ml and 5 μg/ml puromycin respectively.

### *In vitro* migration and invasion assays

3 × 10^4^ SW480 or HCT116 cells in serum-free media were seeded in the upper chambers of Transwell inserts (coated with Matrigel for the invasion assay). Media containing 10% FBS was placed in the lower chamber. After 24 h of incubation, the cells remaining on the membrane upper surface were removed, and the cells that had migrated or invaded through the membrane were fixed in anhydrous methanol and stained with 0.2% crystal violet solution. The migration or invasion activity of the cells was evaluated by the counting cells under an inverted microscope at ×100 magnification. For every chamber, at least 5 fields of view covering the center and periphery of the membrane were assessed. The cell number per field is the mean cell number for the 5 fields (± standard deviation). Differences between cell lines were analyzed using the Student's *t*-test.

### Cell proliferation assay

Cell proliferation rate was determined with Cell Counting Kit-8 (CCK-8) according to the manufacturer's instructions. Briefly, Cells were trypsinized and resuspended in complete medium and plated on 96-well plates (SW480 at 4000 cells/well, HCT116 at 2000 cells/well). At 24, 48, 72 h after incubation, 10 μL of CCK-8 solution was added and mixed well with medium, followed by incubation in the dark for 2 h. Absorbance at 450 nm was then measured on a microplate spectrophotometer (Varioskan LUX, Thermo Scientific).

### Patients and cancer tissues

A total of 192 patients diagnosed with Stage III CRC in Peking Union Medical College Hospital (PUMCH, Beijing, China) were recruited to this study consecutively from 2008 to 2012. None of the patients had chemo- or radiation therapy before “curative” surgery. After surgical excision, CRC tissues were washed thoroughly with ice-cold phosphate buffered saline (PBS) and divided for liquid nitrogen freezing and formalin fixation – paraffin embedding separately.

The medical history and the post-surgical physical examination information of the patients were obtained from the CRC Surveillance Program of the Division of General Surgery of PUMCH. This includes determination of carcinoembryonic antigen-related cell adhesion molecule 5 (CEA) every 3 months for the first 3 years and every 6 months in years 4 and 5 after surgery, colonoscopy in the first year and every 3-5 years thereafter, and other examinations such as a chest X-ray, abdominal ultrasound or CT scans of the chest and abdomen every 6 months for the first 5 years and annually in the sixth to tenth years after diagnosis. Patient data were collected retrospectively through chart review. Complete follow-up, ranging from 2.1 to 84.3 months, was available for all patients and the mean survival time was 42.9 months. At the time of censoring the data, 49/192 (25.5%) patients had died.

The study was performed with the informed consent of the patients and the approval of the Ethics Committee of PUMCH.

### TMT labeling

Cancer tissues were ground in liquid nitrogen and solubilized in lysis buffer (8 M urea in PBS, pH 8.0) containing protease inhibitors. After incubation on ice for 30 min, the pellets were spun down and discarded. The supernatant protein concentration was determined via BCA method.

Proteins were reduced and alkylated with dithiothreitol (DTT) and idoacetamide (IAA), and then diluted with seven-fold volume of PBS. Digestion with Lys-C and trypsin followed the manufacturer's protocol and the reaction was quenched by heating.

Digested proteins were desalted, dried and finally solved in 200 mM triethylammonium bicarbonate buffer. TMT labeling was performed using TMT Mass Tagging Kit following the manufacturer's protocol. Different TMT labels were used to label the different samples in each group, as shown in Table 1. In group IIIB, TMT-126 labeled the G1 sample; TMT-127 for the G2 sample; TMT-128 for the P1 sample; and TMT-130 for the P2 sample. In group IIIC, TMT-126 labeled the G3 sample; TMT-128 for the G4 sample; TMT-130 for the P3 sample; and TMT-131 for the P4 sample.

After labeling, each group samples were pooled, dried and solved in 0.1% trifluoroacetic acid (TFA). The solved two samples were desalted and dried again, and finally solved in 100 μl of 0.1% TFA separately.

Each pooled TMT-labeled samples were further fractionated into 50 fractions using an Xbridge BEH300 C18 column on a Thermo UltiMate 3000 UPLC workstation. Based on the peptide abundance of each fraction, the fractions were combined into 20 samples, dried, and finally solved in 0.1% formic acid for MS analysis.

### Mass spectrometry

LC-MS/MS was performed as described previously [28] with slight modifications. Briefly, the samples were resolved using an Acclaim PepMap RSLC column on a Thermo Scientific UltiMate 3000 RSLCnano System. The eluate was online electrosprayed and analyzed using a Thermo Scientific Q Exactive Hybrid Quadrupole-Orbitrap Mass Spectrometer in positive-ion mode. The MS data from a single full-scan mass spectrum in Orbitrap (350-1,500 *m/z*, 60,000 resolution) followed by a Top10 data-dependent MS/MS scan at 27% high-energy collision-induced dissociation were collected using Thermo Scientific Xcalibur 2.1.2 software in data acquisition mode.

### Data analysis

Protein identification and TMT-based quantification were performed using Proteome Discoverer 1.4 software (Thermo Scientific). In detail, the spectra were extracted from raw MS data files and searched against the Swiss-Prot reviewed human proteome database (downloaded on July 18, 2015, number of protein entries = 20,207) using the Sequest HT algorithm. Precursor Mass Tolerance was 20 ppm, Fragment Mass Tolerance was 0.02 Da and a maximum of two missed cleavages was allowed. Total Intensity Threshold was 20,000 and Minimum Peak Count was 200. Carbamidomethylation (on C) and TMT 6plex (on K and peptide N terminal) were set as static modification, and oxidation (on M) was set as dynamic modification. Protein identification was considered valid if at least one peptide was statistically significant (with a false discovery rate (FDR) of 5%). Default values were used for all other parameters not mentioned above.

TMT 6plex was chosen as the quantification method. Reporter monoisotopic *m/z* was tuned according to the raw spectra data. Proteins were quantified based on only the unique peptide ratio. Protein relative abundances are presented as the ratios to TMT-126.

Search results were read using Proteome Discoverer 1.4 with high peptide confidence filter. To compare protein abundance between patients, the ratios of the protein ACTB were used for normalization between each patient.

The differential expression threshold was defined as 1.5-fold change. As samples from two patients with a good outcome and two patients who suffered distant recurrence were present in each quantification assay, DEPs associated with distant recurrence were defined as proteins whose relative abundances of the two distant recurrence patients were both at least 1.5-fold greater than any one of the good outcome patients.

The mass spectrometry proteomics data have been deposited to the ProteomeXchange Consortium [29] via the PRIDE partner repository with the dataset identifier PXD002903.

### Over-representation analysis and induced interaction network construction

Over-representation analysis and induced interaction network construction were performed using the ConsensusPathDB server [30].

The *p*-value cutoff for over-representation analysis was set as 0.01. For multiple testing, the *p*-values were further corrected by FDR; the level of significance for *q*-values was set as <0.001.

Induced interaction networks were constructed based on “protein interactions”, “genetic interactions”, “biochemical reactions” and “gene regulatory interactions”. For “protein interactions”, only binary interactions were considered. All disconnected protein nodes were removed from the demonstration. The networks were downloaded as tab-delimited text files, and further visualized and re-organized using Cytoscape 3.2.1 [31]. The protein fold change is defined as a ratio of relative abundance average of distant recurrence patients to that of good outcome patients.

### IHC

Briefly, 4 μm-thick tissue sections were dewaxed in xylene and rehydrated in alcohol. For antigen retrieval, sections were incubated in 0.3% hydrogen peroxide solution for 15 min, heated in citrate buffer (pH 6.0) at 95°C for 10 min and cooled to room temperature. The sections were blocked using 10% normal goat serum for 30 min and incubated with diluted primary antibodies overnight at 4°C. Then, the sections were incubated with peroxidase-conjugated secondary antibody and reacted with diaminobenzidine reagent. For negative controls, the primary antibody was replaced with normal rabbit serum. Gastric cancer tissue sections were used as a positive control. Immunoreactivity was evaluated independently by two pathologists. Negative expression was defined as no or weak staining, or staining in less than 30% of all tumor cells regardless of the staining intensity. Positive expression was defined as moderate to strong staining in at least 30% of all tumor cells.

### Statistical analysis

Comparisons of relative protein abundances were performed using the *t*-test module of GraphPad Prism 6 (GraphPad Software, San Diego, CA). *P*-values less than 0.05 were considered statistically significant.

Clinicopathological statistical analysis was performed using SPSS 17.0 (SPSS Inc., Chicago, IL). Unpaired *t*-tests were used to compare differences in time-independent continuous variates, and the chi-square test was used for categorical data. OS and DFS were analyzed using Kaplan–Meier product limit estimator. For univariate analyses, the log-rank test was used to identify prognostic factors. In multivariate analysis, a Cox proportional hazards model was used for all factors found to be significant in univariate analysis, with *p* < 0.05 considered statistically significant.

## SUPPLEMENTARY MATERIALS FIGURES AND TABLES









## Abbreviations

CRC: colorectal cancer
DEP: differentially expressed protein
MS: mass spectrometry
IHC: immunohistochemistry
OS: overall survival
DFS: disease-free survival
GO: Gene Ontology
PUMCH: Peking Union Medical College Hospital
TNM: tumor node metastasis
AJCC: American Joint Committee on Cancer
CEA: carcinoembryonic antigen-related cell adhesion molecule 5
DTT: dithiothreitol
IAA: idoacetamide
TFA: trifluoroacetic acid
FDR: false discovery rate
HR: hazard ratio
CI: confidence interval
