# Supplementary material for: Quantitative proteomics reveals that distant recurrence-associated protein R-Ras and Transgelin predict post-surgical survival in patients with Stage III colorectal cancer
Source: Oncotarget. 2016 May 30;7(28):43868–93. doi: 10.18632/oncotarget.9701 (PMC5190065; doi:10.18632/oncotarget.9701)
Supplement: Supplementary file 1 [file oncotarget-07-43868-s001.pdf]

Quantitative proteomics reveals that distant recurrence-associated protein R-Ras and Transgelin predict post-surgical survival in patients with Stage III colorectal cancer

SUPPLEMENTARY DATA

REFERENCES

1. Bassorgun, C.I, et al., S100A8 and S100A9 Positive Cells in Colorectal Carcinoma: Clinicopathological Analysis. Gastroenterol Res Pract, 2014. 2014:p. 943175.
2. Duan, L., et al., S100A8 and S100A9 are associated with colorectal carcinoma progression and contribute to colorectal carcinoma cell survival and migration via Wnt/beta-catenin pathway. PLoS One, 2013. 8:p. e62092.
3. Kim, H.J., et al., Identification of S100A8 and S100A9 as serological markers for colorectal cancer. J Proteome Res, 2009. 8:p. 1368-79.
4. Kim, J.H., et al., The role of myofibroblasts in upregulation of S100A8 and S100A9 and the differentiation of myeloid cells in the colorectal cancer microenvironment. Biochem Biophys Res Commun, 2012. 423:p. 60-6.
5. Krasnov, G.S., et al., Identification of proteins with altered expression in colorectal cancer by means of 2D-proteomics. Molecular Biology, 2009. 43:p. 321-328.
6. Spitzner, M., et al., A gene expression signature for chemoradiosensitivity of colorectal cancer cells. Int J Radiat Oncol Biol Phys, 2010. 78:p. 1184-92.
7. Wilding, J.L., et al., Replication error deficient and proficient colorectal cancer gene expression differences caused by 3'UTR polyT sequence deletions. Proc Natl Acad Sci U S A, 2010. 107:p. 21058-63.
8. Aychek, T., et al., E-selectin regulates gene expression in metastatic colorectal carcinoma cells and enhances HMGB1 release. Int J Cancer, 2008. 123:p. 1741-50.
9. Lee, H., et al., Diagnostic significance of serum HMGB1 in colorectal carcinomas. PLoS One, 2012. 7:p. e34318.
10. Li, W., et al., HMGB1 recruits myeloid derived suppressor cells to promote peritoneal dissemination of colon cancer after resection. Biochem Biophys Res Commun, 2013. 436:p. 156-61.
11. Liu, W., et al., HMGB1-mediated autophagy modulates sensitivity of colorectal cancer cells to oxaliplatin via MEK/ERK signaling pathway. Cancer Biol Ther, 2015. 16:p. 511-7.
12. Luo, Y., et al., HMGB1 attenuates anti-metastatic defence of the liver in colorectal cancer. Eur J Cancer, 2010. 46:p. 791-9.
13. Moriwaka, Y., et al., HMGB1 attenuates anti-metastatic defense of the lymph nodes in colorectal cancer. Pathobiology, 2010. 77:p. 17-23.
14. Suren, D., et al., The role of high mobility group box 1 (HMGB1) in colorectal cancer. Med Sci Monit, 2014. 20: p. 530-7.
15. Ueda, M., et al., Prognostic significance of high mobility group box 1 (HMGB1) expression in patients with colorectal cancer. Anticancer Res, 2014. 34:p. 5357-62.
16. Yao, X., et al., Overexpression of high-mobility group box 1 correlates with tumor progression and poor prognosis in human colorectal carcinoma. J Cancer Res Clin Oncol, 2010. 136:p. 677-84.
17. Zhang, C.C., et al., The HMGB1 protein sensitizes colon carcinoma cells to cell death triggered by pro-apoptotic agents. Int J Oncol, 2015. 46:p. 667-76.
18. Zhang, Z., et al., Increased HMGB1 and cleaved caspase-3 stimulate the proliferation of tumor cells and are correlated with the poor prognosis in colorectal cancer. J Exp Clin Cancer Res, 2015. 34:p. 51.
19. Kang, U.B., et al., Expression profiling of more than 3500 proteins of MSS-type colorectal cancer by stable isotope labeling and mass spectrometry. J Proteomics, 2012. 75:p. 3050-62.
20. Hostetter, R.B., et al., Carcinoembryonic Antigen as a Selective Enhancer of Colorectal-Cancer Metastasis. Journal of the National Cancer Institute, 1990. 82:p. 380-385.
21. Koprowski, H., et al., Specific antigen in serum of patients with colon carcinoma. Science, 1981. 212:p. 53-5.
22. Bruin, S.C., et al., Molecular alterations associated with liver metastases development in colorectal cancer patients. Br J Cancer, 2011. 105:p. 281-7.
23. Pimiento, J.M., et al., Targeting CSE1L in colorectal cancer. Journal of the American College of Surgeons, 2012. 215:p. S127-S127.
24. Puerta-Garcia, E., M. Canadas-Garre, M.A. Calleja-Hernandez, Molecular biomarkers in colorectal carcinoma. Pharmacogenomics, 2015:p. 1-33.

25. Sethi, M.K., et al., Quantitative proteomic analysis of paired colorectal cancer and non-tumorigenic tissues reveals signature proteins and perturbed pathways involved in CRC progression and metastasis. J Proteomics, 2015. 126:p. 54-67.
26. Tai, C.J., et al., Correlations between cytoplasmic CSE1L in neoplastic colorectal glands and depth of tumor penetration and cancer stage. J Transl Med, 2013. 11:p. 29.
27. Meisenberg, C., et al., Clinical and cellular roles for TDP1 and TOP1 in modulating colorectal cancer response to irinotecan. Mol Cancer Ther, 2015. 14:p. 575-85.
28. Romer, M.U., et al., TOP1 gene copy numbers in colorectal cancer samples and cell lines and their association to *in vitro* drug sensitivity. Scand J Gastroenterol, 2012. 47:p. 68-79.
29. Romer, M.U., et al., Topoisomerase 1(TOP1) gene copy number in stage III colorectal cancer patients and its relation to prognosis. Mol Oncol, 2013. 7:p. 101-11.
30. Smith, D.H., et al., Mechanisms of topoisomerase I (TOP1) gene copy number increase in a stage III colorectal cancer patient cohort. PLoS One, 2013. 8:p. e60613.
31. Sonderstrup, I.M.H., et al., Topoisomerase-1 and-2A gene copy numbers are elevated in mismatch repair-proficient colorectal cancers. Molecular Oncology, 2015. 9:p. 1207-1217.
32. Knol, J.C., et al., Proteomics of differential extraction fractions enriched for chromatin-binding proteins from colon adenoma and carcinoma tissues. Biochim Biophys Acta, 2014. 1844:p. 1034-43.
33. Lu, H., V. Goodell, M.L. Disis, Targeting serum antibody for cancer diagnosis: a focus on colorectal cancer. Expert Opin Ther Targets, 2007. 11:p. 235-44.
34. Staub, E., et al., A genome-wide map of aberrantly expressed chromosomal islands in colorectal cancer. Mol Cancer, 2006. 5:p. 37.
35. Sakai, K., et al., Chemoradiation provides a physiological selective pressure that increases the expansion of aberrant TP53 tumor variants in residual rectal cancerous regions. Oncotarget, 2014. 5:p. 9641-9649.
36. Shin, Y.J., et al., High-mobility group box 2 (HMGB2) modulates radioresponse and is downregulated by p53 in colorectal cancer cell. Cancer Biol Ther, 2013. 14:p. 213-21.
37. Kim, M.S., et al., Expressional analysis of NOLA1, NOLA2, NOLA3 and DKC1, the core proteins in H/ACA riboproteins, in gastric and colorectal cancers. Pathology, 2012. 44:p. 576-7.
38. Choi, D.S., et al., Quantitative proteomics of extracellular vesicles derived from human primary and metastatic colorectal cancer cells. J Extracell Vesicles, 2012. 1.
39. Yan, X.B., et al., Knockdown of Yboxbinding protein1 inhibits the malignant progression of HT29 colorectal adenocarcinoma cells by reversing epithelialmesenchymal transition. Mol Med Rep, 2014. 10:p. 2720-8.
40. Hao, J.M., et al., A five-gene signature as a potential predictor of metastasis and survival in colorectal cancer. J Pathol, 2010. 220:p. 475-89.
41. Gerner, E.W., et al., A comprehensive strategy to combat colon cancer targeting the adenomatous polyposis coli tumor suppressor gene. Ann N Y Acad Sci, 2005. 1059: p. 97-105.
42. Chen, H., et al., Protein-protein interaction analysis of distinct molecular pathways in two subtypes of colorectal carcinoma. Mol Med Rep, 2014. 10:p. 2868-74.
43. Raeisossadati, R., et al., Aberrant expression of DPPA2 and HIWI genes in colorectal cancer and their impacts on poor prognosis. Tumour Biol, 2014. 35:p. 5299-305.
44. Kim, M.S., et al., Frameshift mutations of chromosome cohesion-related genes SGOL1 and PDS5B in gastric and colorectal cancers with high microsatellite instability. Hum Pathol, 2013. 44:p. 2234-40.
45. Deb, S., et al., RAD21 cohesin overexpression is a prognostic and predictive marker exacerbating poor prognosis in KRAS mutant colorectal carcinomas. British Journal of Cancer, 2014. 110:p. 1606-1613.
46. Karl, J., et al., Improved diagnosis of colorectal cancer using a combination of fecal occult blood and novel fecal protein markers. Clin Gastroenterol Hepatol, 2008. 6:p. 1122-8.
47. Thierolf, M., et al., Towards a comprehensive proteome of normal and malignant human colon tissue by 2-D-LC-ESI-MS and 2-DE proteomics and identification of S100A12 as potential cancer biomarker. Proteomics Clin Appl, 2008. 2:p. 11-22.
48. Babel, I., et al., Identification of MST1/STK4 and SULF1 proteins as autoantibody targets for the diagnosis of colorectal cancer by using phage microarrays. Mol Cell Proteomics, 2011. 10:p. M110 001784.
49. Zhai, X.H., et al., Identification of a new protein biomarker for colorectal cancer diagnosis. Mol Med Rep, 2012. 6:p. 444-8.
50. Krzystek-Korpacka, M., et al., Tumor location determines midkine level and its association with the disease progression in colorectal cancer patients: a pilot study. Int J Colorectal Dis, 2012. 27:p. 1319-24.
51. Krzystek-Korpacka, M., et al., Circulating midkine in malignant and non-malignant colorectal diseases. Cytokine, 2013. 64:p. 158-64.
52. Slattery, M.L., et al., Gene expression in colon cancer: A focus on tumor site and molecular phenotype. Genes Chromosomes Cancer, 2015. 54:p. 527-41.
53. Wang, B., et al., A novel all-trans retinoid acid derivatives inhibits the migration of breast cancer cell lines MDA-MB-231 via myosin light chain kinase involving

p38-MAPK pathway. Biomed Pharmacother, 2013. 67:p. 357-62.

54. Ma, Y., et al., Proteomics identification of desmin as a potential oncofetal diagnostic and prognostic biomarker in colorectal cancer. Mol Cell Proteomics, 2009. 8:p. 1878-90.
55. Mikula, M., et al., Integrating proteomic and transcriptomic high-throughput surveys for search of new biomarkers of colon tumors. Functional & Integrative Genomics, 2011. 11:p. 215-224.
56. Yasui, Y., T. Tanaka, Protein expression analysis of inflammation-related colon carcinogenesis. J Carcinog, 2009. 8:p. 10.
57. Park, S.Y., et al., KITENIN-targeting microRNA-124 suppresses colorectal cancer cell motility and tumorigenesis. Mol Ther, 2014. 22:p. 1653-64.
58. Ang, C.S., E.C. Nice, Targeted in-gel MRM: a hypothesis driven approach for colorectal cancer biomarker discovery in human feces. J Proteome Res, 2010. 9:p. 4346-55.
59. Lv, J., et al., Identification of Differentially Expressed Proteins of Normal and Cancerous Human Colorectal Tissues by Liquid Chromatograph-Mass Spectrometer Based on iTRAQ Approach. Cancer Invest, 2015:p. 1-9.
60. Zhu, Y., et al., Gut microbiota and probiotics in colon tumorigenesis. Cancer Lett, 2011. 309:p. 119-27.
61. Ma, Y., et al., Searching for consistently reported up- and down-regulated biomarkers in colorectal cancer: a systematic review of proteomic studies. Mol Biol Rep, 2012. 39:p. 8483-90.
62. Wang, N., et al., Selenium-binding protein 1 is associated with the degree of colorectal cancer differentiation and is regulated by histone modification. Oncol Rep, 2014. 31:p. 2506-14.
63. Lascorz, J., K. Hemminki, A. Forsti, Systematic enrichment analysis of gene expression profiling studies identifies consensus pathways implicated in colorectal cancer development. J Carcinog, 2011. 10:p. 7.
64. Gao, H., et al., Anterior gradient 2: a new target to treat colorectal cancer. Med Hypotheses, 2013. 80:p. 706-8.
65. Riener, M.O., et al., Loss of anterior gradient-2 expression is an independent prognostic factor in colorectal carcinomas. Eur J Cancer, 2014. 50:p. 1722-30.
66. Valladares-Ayerbes, M., et al., Evaluation of the adenocarcinoma-associated gene AGR2 and the intestinal stem cell marker LGR5 as biomarkers in colorectal cancer. Int J Mol Sci, 2012. 13:p. 4367-87.
67. Traicoff, J.L., et al., Characterization of the human polymeric immunoglobulin receptor (PIGR) 3'UTR and differential expression of PIGR mRNA during colon tumorigenesis. J Biomed Sci, 2003. 10:p. 792-804.
68. Bianchini, M., et al., Comparative study of gene expression by cDNA microarray in human colorectal cancer tissues and normal mucosa. Int J Oncol, 2006. 29:p. 83-94.
69. Bongaerts, B.W., et al., Alcohol consumption, alcohol dehydrogenase 1C (ADH1C) genotype, and risk of colorectal cancer in the Netherlands Cohort Study on diet and cancer. Alcohol, 2011. 45:p. 217-25.
70. Yang, H.Y., et al., Comparative proteomic analysis of cysteine oxidation in colorectal cancer patients. Mol Cells, 2013. 35:p. 533-42.
71. Crous-Bou, M., et al., Polymorphisms in alcohol metabolism genes ADH1B and ALDH2, alcohol consumption and colorectal cancer. PLoS One, 2013. 8:p. e80158.
72. Guo, X.F., et al., Meta-analysis of the ADH1B and ALDH2 polymorphisms and the risk of colorectal cancer in East Asians. Intern Med, 2013. 52:p. 2693-9.
73. Gao, Z.H., et al., ILEI: a novel marker for epithelial-mesenchymal transition and poor prognosis in colorectal cancer. Histopathology, 2014. 65:p. 527-38.
74. Lind, G.E., et al., Identification of an epigenetic biomarker panel with high sensitivity and specificity for colorectal cancer and adenomas. Mol Cancer, 2011. 10:p. 85.
75. Takemasa, I., et al., Potential biological insights revealed by an integrated assessment of proteomic and transcriptomic data in human colorectal cancer. Int J Oncol, 2012. 40:p. 551-9.
76. Wang, H.Y., et al., Expression status of S100A14 and S100A4 correlates with metastatic potential and clinical outcome in colorectal cancer after surgery. Oncol Rep, 2010. 23:p. 45-52.
77. Gormley, J.A., et al., The role of Cathepsin S as a marker of prognosis and predictor of chemotherapy benefit in adjuvant CRC: a pilot study. British Journal of Cancer, 2011. 105:p. 1487-1494.
78. Kuester, D., et al., The cathepsin family and their role in colorectal cancer. Pathology Research and Practice, 2008. 204:p. 491-500.
79. Choi, D.S., et al., Proteomic analysis of microvesicles derived from human colorectal cancer ascites. Proteomics, 2011. 11:p. 2745-51.
80. Kim, S.W., et al., Abrogation of galectin-4 expression promotes tumorigenesis in colorectal cancer. Cell Oncol (Dordr), 2013. 36:p. 169-78.
81. Rechreche, H., et al., Cloning and expression of the mRNA of human galectin-4, an S-type lectin down-regulated in colorectal cancer. European Journal of Biochemistry, 1997. 248:p. 225-230.
82. Satelli, A., et al., Galectin-4 functions as a tumor suppressor of human colorectal cancer. International Journal of Cancer, 2011. 129:p. 799-809.
83. Ye, H., et al., Meta-analysis of human colorectal cancer transcriptome. Int J Colorectal Dis, 2012. 27:p. 1125-8.

84. Perilli, L., et al., Circulating miR-182 is a biomarker of colorectal adenocarcinoma progression. Oncotarget, 2014. 5:p. 6611-6619.
85. Pizzini, S., et al., Impact of microRNAs on regulatory networks and pathways in human colorectal carcinogenesis and development of metastasis. BMC Genomics, 2013. 14: p. 589.
86. Groschl, B., et al., Iqgap2 is downregulated in colorectal cancer (crc) and involved in cellular migration. Cancer Research, 2014. 74:p. 5004-5004.
87. Roepman, P., et al., Colorectal cancer intrinsic subtypes predict chemotherapy benefit, deficient mismatch repair and epithelial-to-mesenchymal transition. International Journal of Cancer, 2014. 134:p. 552-562.
88. Zhang, C., et al., Lack of association of SULT1A1 R213H polymorphism with colorectal cancer: a meta-analysis. PLoS One, 2011. 6:p. e19127.
89. Otero-Estevez, O., et al., Decreased Expression of Alpha-L-Fucosidase Gene FUCA1 in Human Colorectal Tumors. International Journal of Molecular Sciences, 2013. 14:p. 16986-16998.
90. Hong, Y., et al., A 'metastasis-prone' signature for early-stage mismatch-repair proficient sporadic colorectal cancer patients and its implications for possible therapeutics. Clinical & Experimental Metastasis, 2010. 27:p. 83-90.
91. Soreide, K., et al., Trypsin in colorectal cancer: molecular biological mechanisms of proliferation, invasion, and metastasis. J Pathol, 2006. 209:p. 147-56.
92. Chen, Q., et al., Microarray analyses reveal liver metastasis-related genes in metastatic colorectal cancer cell model. J Cancer Res Clin Oncol, 2013. 139:p. 1169-78.
93. Fernandez-Rozadilla, C., et al., Pharmacogenomics in colorectal cancer: a genome-wide association study to predict toxicity after 5-fluorouracil or FOLFOX administration. Pharmacogenomics J, 2013. 13:p. 209-17.
94. Gylfe, A.E., et al., Identification of candidate oncogenes in human colorectal cancers with microsatellite instability. Gastroenterology, 2013. 145:p. 540-3 e22.
95. Yeh, C.S., et al., Fatty acid metabolism pathway play an important role in carcinogenesis of human colorectal cancers by Microarray-Bioinformatics analysis. Cancer Lett, 2006. 233:p. 297-308.
96. Yi, J.M., et al., Genomic and epigenomic integration identifies a prognostic signature in colon cancer. Clin Cancer Res, 2011. 17:p. 1535-45.
97. McHugh, S.M., J. O'Donnell, P. Gillen, Genomic and oncoproteomic advances in detection and treatment of colorectal cancer. World J Surg Oncol, 2009. 7:p. 36.
98. Chik, J.H.L., et al., Comprehensive glycomics comparison between colon cancer cell cultures and tumours: Implications for biomarker studies. Journal of Proteomics, 2014. 108:p. 146-162.
99. Schafer, H., et al., TGF-beta 1-dependent L1CAM expression has an essential role in macrophage-induced apoptosis resistance and cell migration of human intestinal epithelial cells. Oncogene, 2013. 32:p. 180-189.
100. Lee, S., et al., Identification of GABRA1 and LAMA2 as new DNA methylation markers in colorectal cancer. International Journal of Oncology, 2012. 40:p. 889-898.
101. Hansen, A.G., et al., Elevated ALCAM shedding in colorectal cancer correlates with poor patient outcome. Cancer Res, 2013. 73:p. 2955-64.
102. Tachezy, M., et al., Activated leukocyte cell adhesion molecule (CD166)-Its prognostic power for colorectal cancer patients. Journal of Surgical Research, 2012. 177: p. E15-E20.
103. Budinska, E., et al., Gene expression patterns unveil a new level of molecular heterogeneity in colorectal cancer. J Pathol, 2013. 231:p. 63-76.
104. Dou, R., et al., Multi-microarray identifies lower AQP9 expression in adjuvant chemotherapy nonresponders with stage III colorectal cancer. Cancer Lett, 2013. 336:p. 106-13.
105. Birkenkamp-Demtroder, K., et al., Keratin23 (KRT23) knockdown decreases proliferation and affects the DNA damage response of colon cancer cells. PLoS One, 2013. 8:p. e73593.
106. Tian, X., et al., Screening of potential diagnostic markers and therapeutic targets against colorectal cancer. Onco Targets Ther, 2015. 8:p. 1691-9.
107. Yeh, C.S., et al., Significance of the glycolytic pathway and glycolysis related-genes in tumorigenesis of human colorectal cancers. Oncol Rep, 2008. 19:p. 81-91.
108. Koike, Y., et al., Preoperative C-Reactive Protein as a Prognostic and Therapeutic Marker for Colorectal Cancer. Journal of Surgical Oncology, 2008. 98:p. 540-544.
109. Shiu, Y.C., et al., Is C-reactive protein a prognostic factor of colorectal cancer? Dis Colon Rectum, 2008. 51:p. 443-9.
110. Tsilidis, K.K., et al., C-reactive protein and colorectal cancer risk: a systematic review of prospective studies. Int J Cancer, 2008. 123:p. 1133-40.
111. Lin, Y., et al., Association of the actin-binding protein transgelin with lymph node metastasis in human colorectal cancer. Neoplasia, 2009. 11:p. 864-73.
112. Peng, J.Y., et al., A rat-to-human search for proteomic alterations reveals transgelin as a biomarker relevant to colorectal carcinogenesis and liver metastasis. Electrophoresis, 2009. 30:p. 2976-2987.

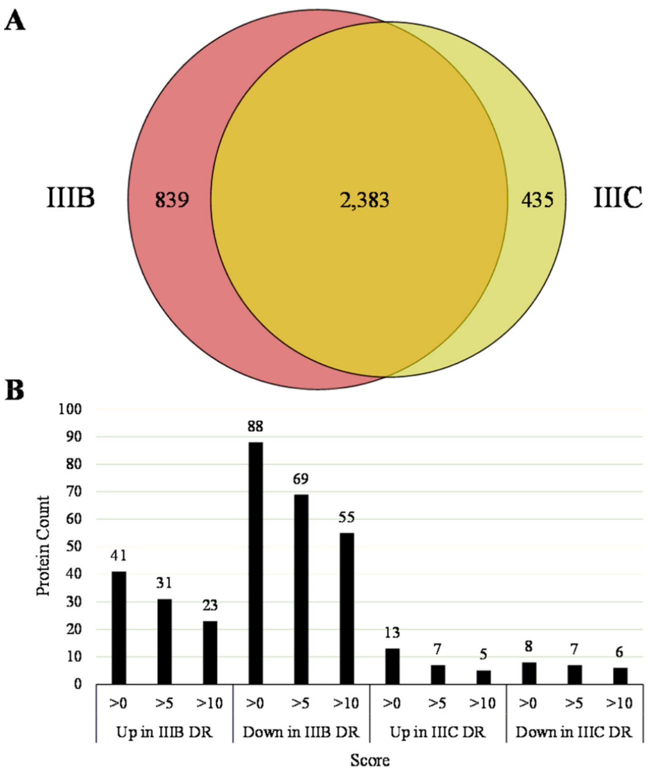

Supplementary Figure S1: Quantitative MS identified 146 DEPs associated with distant recurrence. A. 2,383 proteins were identified in both IIIB and IIIC groups. Additionally, 839 IIIB-specific proteins and 435 IIIC-specific proteins were also identified. **B.** Out of the total 3,657 proteins, 146 proteins were differentially expressed between good outcome and distant recurrence. About half of them had a score more than 10. (DR, distant recurrence).

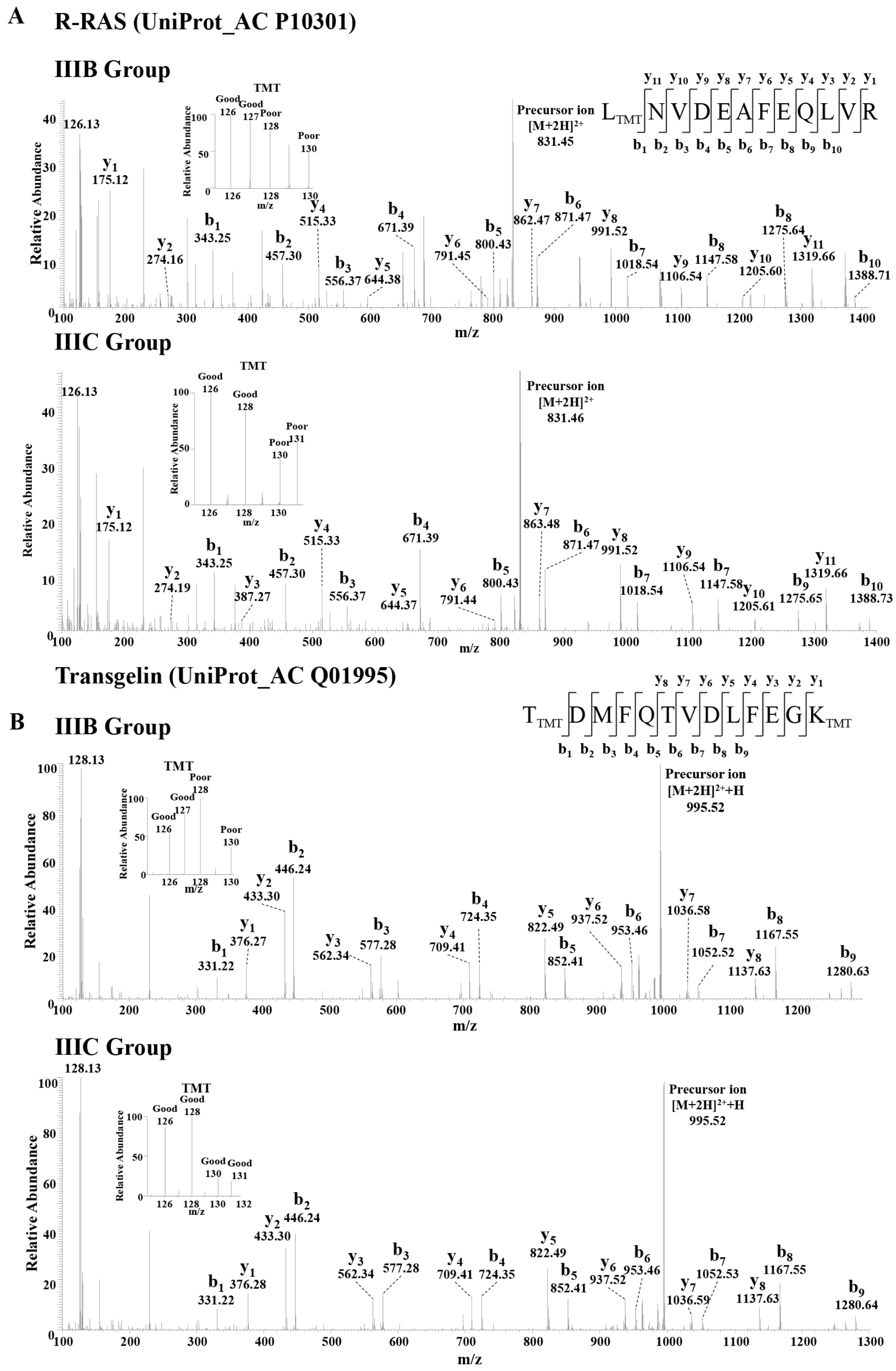

Supplementary Figure S2: MS/MS spectra of R-Ras and Transgelin unique peptides. A. MS/MS spectra of R-Ras aa177-188 (m/z = 831.45) in the MS experiments of IIIB and IIIC groups. **B.** MS/MS spectra of Transgelin aa109-121 (m/z = 995.52) in the MS experiments of IIIB and IIIC groups. The TMT reporter signals were also shown.

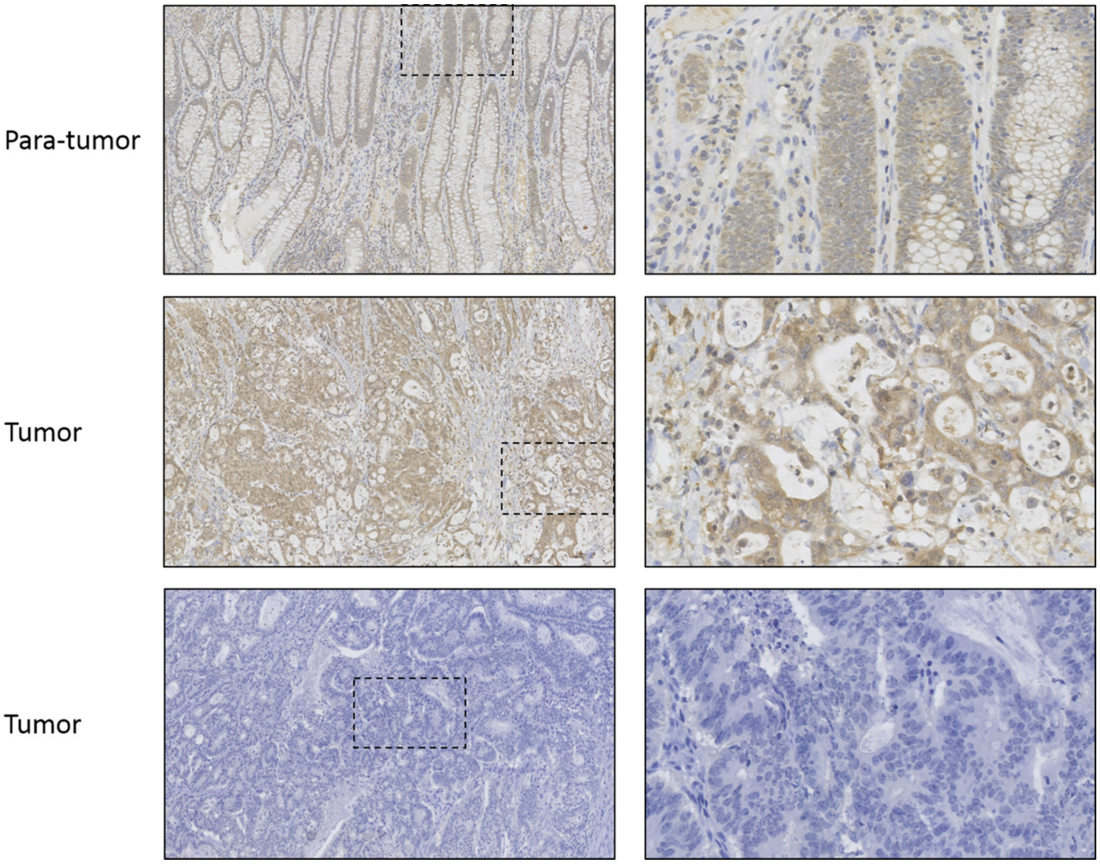

Supplementary Figure S3: Representative photos (amplification: 100) of anti-R-Ras IHC with regions zoomed in (amplification: 400). Top panel: R-Ras dense staining is observed in epithelial cells of longitudinally cut mucosa crypts of para-tumor tissue. Medium panel: positive R-Ras staining of poorly differentiated adenocarcinoma. Bottom panel: well differentiated adenocarcinoma with negative R-Ras staining.

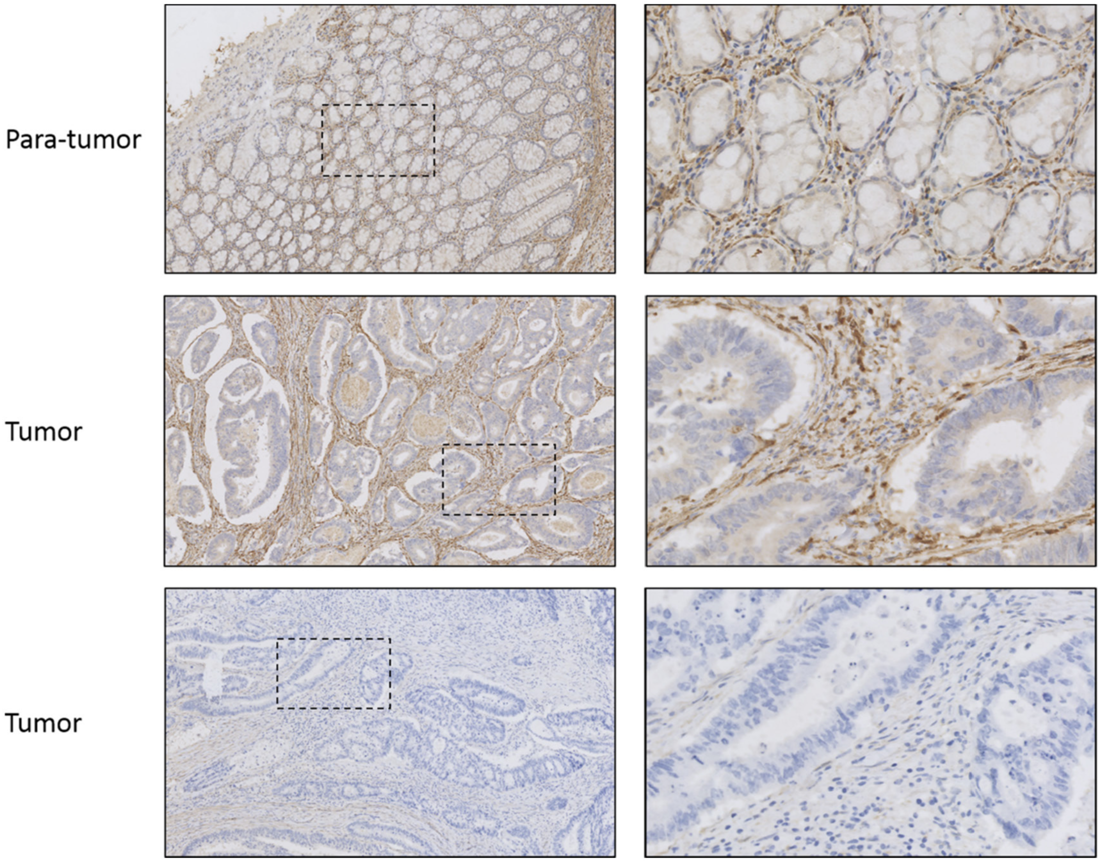

Supplementary Figure S4: Representative photos (amplification: 100) of anti-Transgelin IHC with regions zoomed in (amplification: 400). Top panel: Transgelin dense staining is observed around tangentially cut mucosa crypts of para-tumor tissue. Medium panel: moderately differentiated tumor tissue of positive Transgelin staining. Bottom panel: Transgelin-negative tumor tissue shows desmoplasia. Necrotic debris can be observed in glandular lumina.

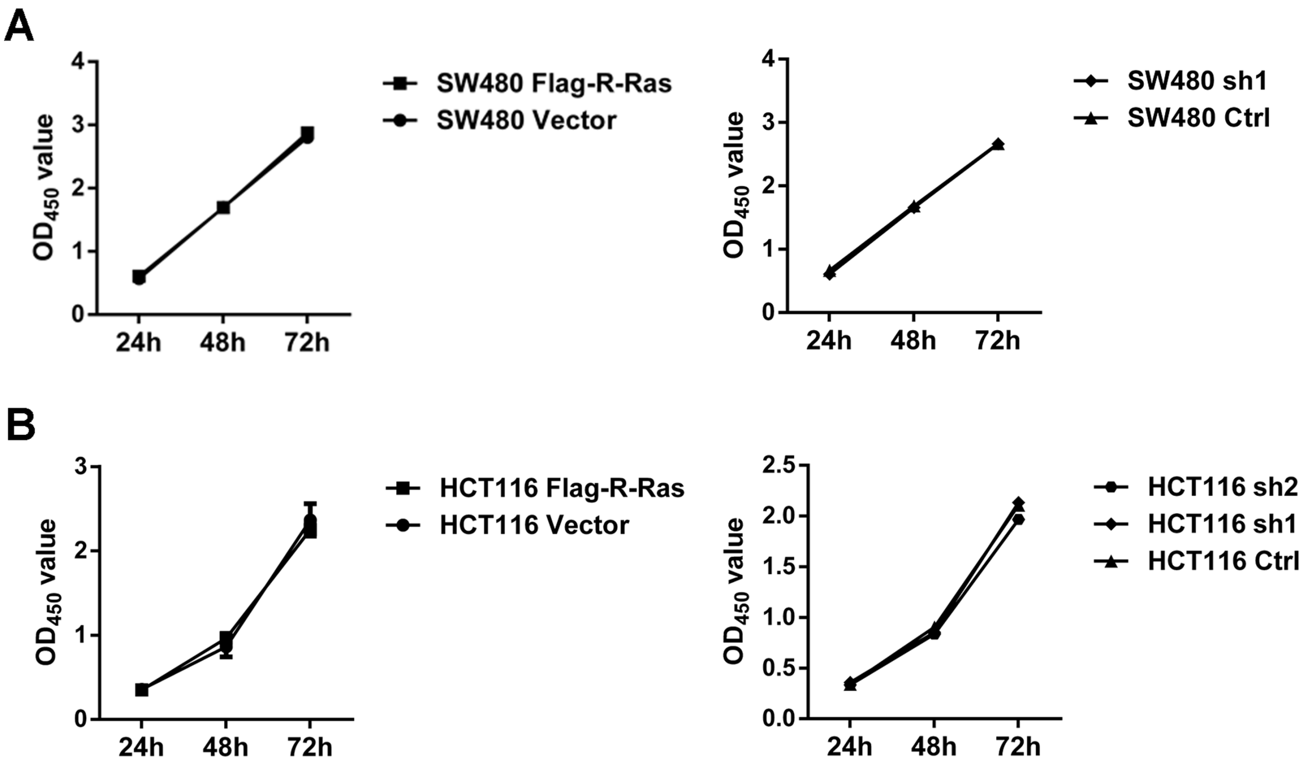

Supplementary Figure S5: R-Ras has no effect on CRC cells proliferation. (A) SW480 and (B) HCT116 were stably transfected with pLv-CP06 vector and pLv-CP06-Flag-RRAS (left panel); pLv-shRNA-KP Ctrl, pLv-shRNA-KP-sh1, and pLv-shRNA-KP-sh2 (right panel), respectively. Monolayer growth rates of cells were determined by CCK8 assay. Results are presented as means±SD (n=4).

Supplementary Table S1:

See Supplementary File 1

Supplementary Table S2: Manual search in literature databases found that the relevance of 66 DEPs with CRC has been reported previously

See Supplementary File 2

Supplementary Table S3: R-Ras and Transgelin expression in tumor and para-tumor tissue of Stage III CRC

Protein	Tissue type	Number	Staining		*p*-value	χ2 value
			Positive	Negative		
R-Ras	Tumor	192	83	109	<0.001	59.21
	Para-tumor	192	157	35		
Transgelin	Tumor	192	84	108	<0.001	75.21
	Para-tumor	192	166	26		

Supplementary Table S4: DEPs expression measurement information IIIB

See Supplementary File 3
