# Supplementary material for: Quantitative proteomics reveals that distant recurrence-associated protein R-Ras and Transgelin predict post-surgical survival in patients with Stage III colorectal cancer
Source: Oncotarget. 2016 May 30;7(28):43868–93. doi: 10.18632/oncotarget.9701 (PMC5190065; doi:10.18632/oncotarget.9701)
Supplement: Supplementary file 3 [file oncotarget-07-43868-s003.docx]

| **Protein** | **Previous reports** |
| --- | --- |
| Protein S100-A8 GN=S100A8 | [1], [2], [3], [4] |
| Heterogeneous nuclear ribonucleoprotein H GN=HNRNPH1 | [5], [6], [7] |
| High mobility group protein B1 GN=HMGB1 | [8], [9], [10], [11], [12], [13], [14], [15], [16], [17], [18] |
| Lactotransferrin GN=LTF | NF |
| Neutrophil defensin 3 GN=DEFA3 | [19] |
| Carcinoembryonic antigen-related cell adhesion molecule 5 GN=CEACAM5 | [20], [21] |
| Exportin-2 GN=CSE1L | [22], [23], [24], [25], [26] |
| DNA topoisomerase 1 GN=TOP1 | [27], [28], [29], [30], [31] |
| Nucleosome assembly protein 1-like 1 GN=NAP1L1 | [32], [33], [34] |
| Sorting nexin-6 GN=SNX6 | NF |
| High mobility group protein B2 GN=HMGB2 | [35], [36] |
| Lamina-associated polypeptide 2, isoforms beta/gamma GN=TMPO | NF |
| Nucleolar protein 58 GN=NOP58 | NF |
| H/ACA ribonucleoprotein complex subunit 2 GN=NHP2 | [37] |
| rRNA 2'-O-methyltransferase fibrillarin GN=FBL | NF |
| Nuclease-sensitive element-binding protein 1 GN=YBX1 | [38], [39] |
| Probable global transcription activator SNF2L2 GN=SMARCA2 | [40] |
| Ran-specific GTPase-activating protein GN=RANBP1 | NF |
| Ribosome maturation protein SBDS GN=SBDS | NF |
| DCN1-like protein 1 GN=DCUN1D1 | NF |
| Rab11 family-interacting protein 1 GN=RAB11FIP1 | NF |
| Cellular nucleic acid-binding protein GN=CNBP | [41] |
| Fragile X mental retardation syndrome-related protein 2 GN=FXR2 | [42], [43] |
| Eukaryotic translation initiation factor 1A, Y-chromosomal GN=EIF1AY | NF |
| Sister chromatid cohesion protein PDS5 homolog B GN=PDS5B | [44] |
| Pinin GN=PNN | NF |
| Cancer-related nucleoside-triphosphatase GN=NTPCR | NF |
| Double-strand-break repair protein rad21 homolog GN=RAD21 | [45] |
| Aggrecan core protein GN=ACAN | NF |
| Endothelial differentiation-related factor 1 GN=EDF1 | NF |
| Protein S100-A12 GN=S100A12 | [46], [47] |
| Serine/threonine-protein kinase 4 GN=STK4 | [48], [49] |
| Cytochrome c oxidase subunit NDUFA4 GN=NDUFA4 | NF |
| Midkine GN=MDK | [50], [51] |
| Cytochrome c oxidase subunit 2 GN=MT-CO2 | NF |
| Tetratricopeptide repeat protein 1 GN=TTC1 | NF |
| Putative hexokinase HKDC1 GN=HKDC1 | [52] |
| Golgi SNAP receptor complex member 2 GN=GOSR2 | NF |
| Putative ATP-dependent RNA helicase DHX57 GN=DHX57 | NF |
| FAD-dependent oxidoreductase domain-containing protein 1 GN=FOXRED1 | NF |
| Sodium/glucose cotransporter 5 GN=SLC5A10 | NF |
| Myosin-11 GN=MYH11 | [53] |
| Desmin GN=DES | [54] |
| Ig alpha-2 chain C region GN=IGHA2 | NF |
| Creatine kinase B-type GN=CKB | [5] |
| IgGFc-binding protein GN=FCGBP | [55], [56] |
| Collagen alpha-1(XIV) chain GN=COL14A1 | NF |
| Myosin regulatory light polypeptide 9 GN=MYL9 | [57] |
| Filamin-C GN=FLNC | [58], [59] |
| Mucin-2 GN=MUC2 | [60] |
| Selenium-binding protein 1 GN=SELENBP1 | [61], [62] |
| Carbonic anhydrase 1 GN=CA1 | [63] |
| Anterior gradient protein 2 homolog GN=AGR2 | [64], [65], [66] |
| Polymeric immunoglobulin receptor GN=PIGR | [67] |
| Intelectin-1 GN=ITLN1 | NF |
| Ig mu chain C region GN=IGHM | [68] |
| Sialic acid synthase GN=NANS | NF |
| Alcohol dehydrogenase 1C GN=ADH1C | [69] |
| Tryptase alpha/beta-1 GN=TPSAB1 | [19], [70] |
| Alcohol dehydrogenase 1B GN=ADH1B | [71], [72] |
| Trefoil factor 3 GN=TFF3 | NF |
| Carbonic anhydrase 2 GN=CA2 | [63] |
| Laminin subunit beta-2 GN=LAMB2 | NF |
| Protein FAM3D GN=FAM3D | [73] |
| Granulins GN=GRN | NF |
| Four and a half LIM domains protein 1 GN=FHL1 | [74], [75] |
| ATP synthase subunit delta, mitochondrial GN=ATP5D | NF |
| Phosphoglucomutase-like protein 5 GN=PGM5 | NF |
| Protein S100-A14 GN=S100A14 | [76] |
| Aldose 1-epimerase GN=GALM | NF |
| Cathepsin S GN=CTSS | [77], [78] |
| Immunoglobulin J chain GN=IGJ | NF |
| Galectin-4 GN=LGALS4 | [79], [80], [81], [82] |
| Acetyl-CoA acetyltransferase, mitochondrial GN=ACAT1 | NF |
| Chymase GN=CMA1 | NF |
| Zymogen granule membrane protein 16 GN=ZG16 | [83] |
| Acyl-coenzyme A thioesterase 1 GN=ACOT1 | NF |
| Alpha-amylase 1 GN=AMY1A | NF |
| Eosinophil cationic protein GN=RNASE3 | NF |
| Hydroxyacyl-coenzyme A dehydrogenase, mitochondrial GN=HADH | NF |
| Ectonucleoside triphosphate diphosphohydrolase 5 GN=ENTPD5 | [55], [84], [85] |
| Beta-hexosaminidase subunit alpha GN=HEXA | NF |
| Adseverin GN=SCIN | NF |
| WAP four-disulfide core domain protein 2 GN=WFDC2 | NF |
| Ras GTPase-activating-like protein IQGAP2 GN=IQGAP2 | [86] |
| Carcinoembryonic antigen-related cell adhesion molecule 7 GN=CEACAM7 | [25] |
| ATP synthase-coupling factor 6, mitochondrial GN=ATP5J | NF |
| Aflatoxin B1 aldehyde reductase member 3 GN=AKR7A3 | NF |
| Short/branched chain specific acyl-CoA dehydrogenase, mitochondrial GN=ACADSB | [87] |
| Sulfotransferase 1A1 GN=SULT1A1 | [88] |
| Spondin-1 GN=SPON1 | NF |
| Guanine nucleotide-binding protein G(o) subunit alpha GN=GNAO1 | NF |
| Dehydrogenase/reductase SDR family member 11 GN=DHRS11 | NF |
| Tissue alpha-L-fucosidase GN=FUCA1 | [89] |
| Matrilin-2 GN=MATN2 | [90] |
| Adapter molecule crk GN=CRK | NF |
| Trypsin-1 GN=PRSS1 | [91] |
| Ras-related protein R-Ras GN=RRAS | NF |
| Sialate O-acetylesterase GN=SIAE | NF |
| Carboxymethylenebutenolidase homolog GN=CMBL | NF |
| Isochorismatase domain-containing protein 1 GN=ISOC1 | NF |
| Integrin alpha-7 GN=ITGA7 | [92], [93], [94], [19] |
| NADH dehydrogenase [ubiquinone] iron-sulfur protein 4, mitochondrial GN=NDUFS4 | NF |
| Short-chain specific acyl-CoA dehydrogenase, mitochondrial GN=ACADS | [95] |
| Hyaluronan and proteoglycan link protein 1 GN=HAPLN1 | [96] |
| Target of Nesh-SH3 GN=ABI3BP | NF |
| Secretagogin GN=SCGN | [97] |
| Heme oxygenase 1 GN=HMOX1 | NF |
| Alpha-N-acetylgalactosaminide alpha-2,6-sialyltransferase 1 GN=ST6GALNAC1 | [98] |
| Stromal cell-derived factor 2-like protein 1 GN=SDF2L1 | NF |
| Centrosomal protein of 131 kDa GN=CEP131 | NF |
| Glucagon GN=GCG | NF |
| Syndecan-1 GN=SDC1 | NF |
| N(4)-(beta-N-acetylglucosaminyl)-L-asparaginase GN=AGA | NF |
| Sulfotransferase family cytosolic 1B member 1 GN=SULT1B1 | [19] |
| Neural cell adhesion molecule L1 GN=L1CAM | [99] |
| Chromogranin-A GN=CHGA | NF |
| Tropomodulin-1 GN=TMOD1 | NF |
| Laminin subunit alpha-2 GN=LAMA2 | [100] |
| CD166 antigen GN=ALCAM | [101], [102] |
| Carbonic anhydrase 12 GN=CA12 | [63] |
| UDP-GlcNAc:betaGal beta-1,3-N-acetylglucosaminyltransferase 7 GN=B3GNT7 | [103], [104] |
| RNA-binding protein 47 GN=RBM47 | NF |
| Thrombospondin type-1 domain-containing protein 4 GN=THSD4 | NF |
| Kallikrein-1 GN=KLK1 | NF |
| Charged multivesicular body protein 6 GN=CHMP6 | NF |
| Mannose-1-phosphate guanyltransferase alpha GN=GMPPA | NF |
| Dihydropteridine reductase GN=QDPR | NF |
| Placenta-specific protein 9 GN=PLAC9 | NF |
| Ig gamma-3 chain C region GN=IGHG3 | NF |
| Prosaposin GN=PSAP | NF |
| Beta-hexosaminidase subunit beta GN=HEXB | NF |
| Histone H1.0 GN=H1F0 | NF |
| Keratin, type I cytoskeletal 23 GN=KRT23 | [105] |
| 60S ribosomal protein L36a GN=RPL36A | [106] |
| HLA class II histocompatibility antigen, DRB1-14 beta chain GN=HLA-DRB1 | NF |
| Fructose-bisphosphate aldolase B GN=ALDOB | [107] |
| Polypyrimidine tract-binding protein 3 GN=PTBP3 | NF |
| C-reactive protein GN=CRP | [108], [109], [110] |
| Latexin GN=LXN | NF |
| ABI gene family member 3 GN=ABI3 | NF |
| Actin, aortic smooth muscle GN=ACTA2 | NF |
| Transgelin GN=TAGLN | [111], [112] |
| Tropomyosin beta chain GN=TPM2 | NF |
| Synemin GN=SYNM | NF |
| Signal peptidase complex subunit 2 GN=SPCS2 | NF |

**Supplementary Table S2** Manual search in literature databases found that the relevance of 66 DEPs with CRC has been reported previously (GN, gene name, NF, not found).
