# Supplementary material for: Quantitative proteomics reveals that distant recurrence-associated protein R-Ras and Transgelin predict post-surgical survival in patients with Stage III colorectal cancer
Source: Oncotarget. 2016 May 30;7(28):43868–93. doi: 10.18632/oncotarget.9701 (PMC5190065; doi:10.18632/oncotarget.9701)
Supplement: Supplementary file 4 [file oncotarget-07-43868-s004.docx]

**IIIB**

| **Accession** | **Relative Abundance in IIIB Group** | | | | **Fold Change** | **log_1.5_(fold change)** |
| --- | --- | --- | --- | --- | --- | --- |
|  | **126-G1** | **127-G2** | **128-P1** | **130-P2** |  |  |
| P05109 | 1.000 | 0.919 | 1.504 | 1.572 | 1.603 | 1.164 |
| P31943 | 1.000 | 1.061 | 1.822 | 1.918 | 1.815 | 1.470 |
| P09429 | 1.000 | 0.857 | 2.124 | 1.689 | 2.054 | 1.775 |
| P02788 | 1.000 | 0.870 | 1.853 | 1.572 | 1.831 | 1.492 |
| P59666 | 1.000 | 0.883 | 1.808 | 1.585 | 1.802 | 1.452 |
| P06731 | 1.000 | 0.877 | 1.558 | 1.849 | 1.815 | 1.471 |
| P55060 | 1.000 | 1.095 | 1.888 | 2.108 | 1.907 | 1.592 |
| P11387 | 1.000 | 0.917 | 1.505 | 1.649 | 1.645 | 1.228 |
| P55209 | 1.000 | 1.319 | 2.020 | 3.629 | 2.436 | 2.196 |
| Q9UNH7 | 1.000 | 1.191 | 2.039 | 1.887 | 1.792 | 1.439 |
| P26583 | 1.000 | 1.023 | 2.260 | 1.947 | 2.080 | 1.806 |
| P42167 | 1.000 | 0.997 | 1.981 | 1.785 | 1.886 | 1.565 |
| Q9Y2X3 | 1.000 | 1.014 | 1.676 | 1.624 | 1.638 | 1.218 |
| Q9NX24 | 1.000 | 1.176 | 2.419 | 1.997 | 2.029 | 1.745 |
| P22087 | 1.000 | 0.964 | 1.769 | 1.685 | 1.758 | 1.392 |
| P67809 | 1.000 | 0.957 | 1.904 | 1.948 | 1.968 | 1.670 |
| P51531 | 1.000 | 0.953 | 2.580 | 1.701 | 2.192 | 1.936 |
| P43487 | 1.000 | 1.099 | 2.060 | 2.162 | 2.011 | 1.723 |
| Q9Y3A5 | 1.000 | 1.078 | 1.728 | 1.664 | 1.632 | 1.208 |
| Q96GG9 | 1.000 | 0.911 | 1.597 | 1.522 | 1.631 | 1.207 |
| Q6WKZ4 | 1.000 | 1.103 | 2.071 | 2.002 | 1.937 | 1.630 |
| P62633 | 1.000 | 1.255 | 3.139 | 2.075 | 2.312 | 2.067 |
| P51116 | 1.000 | 1.013 | 1.606 | 1.604 | 1.594 | 1.150 |
| O14602 | 1.000 | 1.168 | 2.391 | 2.128 | 2.084 | 1.811 |
| Q9NTI5 | 1.000 | 1.138 | 1.767 | 2.287 | 1.896 | 1.578 |
| Q9H307 | 1.000 | 1.266 | 2.646 | 3.236 | 2.596 | 2.352 |
| Q9BSD7 | 1.000 | 1.003 | 2.052 | 1.720 | 1.883 | 1.561 |
| O60216 | 1.000 | 1.039 | 2.119 | 1.595 | 1.821 | 1.478 |
| P16112 | 1.000 | 1.045 | 2.404 | 2.099 | 2.202 | 1.947 |
| O60869 | 1.000 | 1.210 | 2.379 | 2.868 | 2.374 | 2.132 |
| P80511 | 1.000 | 0.741 | 7.672 | 1.707 | 5.387 | 4.153 |
| Q13043 | 1.000 | 0.987 | 1.807 | 1.559 | 1.695 | 1.301 |
| O00483 | 1.000 | 1.118 | 2.170 | 1.993 | 1.965 | 1.667 |
| P21741 | 1.000 | 0.875 | 1.682 | 1.921 | 1.922 | 1.611 |
| P00403 | 1.000 | 0.849 | 1.569 | 1.643 | 1.737 | 1.362 |
| Q99614 | 1.000 | 1.058 | 2.099 | 1.733 | 1.862 | 1.533 |
| Q2TB90 | 1.000 | 0.963 | 2.355 | 1.938 | 2.187 | 1.929 |
| O14653 | 1.000 | 1.001 | 1.523 | 1.588 | 1.555 | 1.090 |
| Q6P158 | 1.000 | 1.082 | 1.868 | 1.651 | 1.690 | 1.294 |
| Q96CU9 | 1.000 | 1.000 | 1.535 | 1.517 | 1.525 | 1.041 |
| A0PJK1 | 1.000 | 1.026 | 2.543 | 2.031 | 2.258 | 2.009 |
| P35749 | 1.000 | 0.922 | 0.454 | 0.484 | 0.488 | -1.769 |
| P17661 | 1.000 | 1.080 | 0.337 | 0.347 | 0.329 | -2.744 |
| P01877 | 1.000 | 0.734 | 0.310 | 0.336 | 0.373 | -2.433 |
| P12277 | 1.000 | 1.279 | 0.533 | 0.599 | 0.497 | -1.726 |
| Q9Y6R7 | 1.000 | 0.864 | 0.461 | 0.449 | 0.488 | -1.768 |
| Q05707 | 1.000 | 0.944 | 0.478 | 0.413 | 0.458 | -1.925 |
| P24844 | 1.000 | 1.017 | 0.642 | 0.576 | 0.604 | -1.243 |
| Q14315 | 1.000 | 0.926 | 0.615 | 0.616 | 0.639 | -1.104 |
| Q02817 | 1.000 | 1.142 | 0.434 | 0.474 | 0.424 | -2.118 |
| Q13228 | 1.000 | 1.430 | 0.415 | 0.501 | 0.377 | -2.405 |
| P00915 | 1.000 | 1.233 | 0.463 | 0.513 | 0.437 | -2.042 |
| O95994 | 1.000 | 1.038 | 0.491 | 0.551 | 0.511 | -1.655 |
| P01833 | 1.000 | 0.832 | 0.305 | 0.334 | 0.349 | -2.599 |
| Q8WWA0 | 1.000 | 0.516 | 0.264 | 0.270 | 0.352 | -2.572 |
| P01871 | 1.000 | 0.870 | 0.525 | 0.513 | 0.555 | -1.452 |
| Q9NR45 | 1.000 | 1.071 | 0.611 | 0.611 | 0.590 | -1.302 |
| P00326 | 1.000 | 0.806 | 0.394 | 0.441 | 0.463 | -1.901 |
| Q15661 | 1.000 | 0.910 | 0.386 | 0.496 | 0.462 | -1.904 |
| P00325 | 1.000 | 0.878 | 0.503 | 0.491 | 0.529 | -1.570 |
| Q07654 | 1.000 | 0.893 | 0.458 | 0.456 | 0.482 | -1.798 |
| P00918 | 1.000 | 1.659 | 0.477 | 0.555 | 0.388 | -2.335 |
| P55268 | 1.000 | 1.081 | 0.644 | 0.613 | 0.604 | -1.243 |
| Q96BQ1 | 1.000 | 0.981 | 0.343 | 0.415 | 0.382 | -2.372 |
| P28799 | 1.000 | 0.944 | 0.597 | 0.526 | 0.578 | -1.354 |
| Q13642 | 1.000 | 1.122 | 0.631 | 0.619 | 0.589 | -1.306 |
| P30049 | 1.000 | 0.926 | 0.604 | 0.520 | 0.583 | -1.329 |
| Q15124 | 1.000 | 0.772 | 0.417 | 0.406 | 0.465 | -1.890 |
| Q9HCY8 | 1.000 | 0.933 | 0.524 | 0.516 | 0.538 | -1.530 |
| Q96C23 | 1.000 | 1.088 | 0.473 | 0.598 | 0.513 | -1.646 |
| P25774 | 1.000 | 0.978 | 0.647 | 0.652 | 0.656 | -1.038 |
| P01591 | 1.000 | 0.779 | 0.499 | 0.455 | 0.537 | -1.536 |
| P56470 | 1.000 | 0.829 | 0.493 | 0.479 | 0.531 | -1.560 |
| P24752 | 1.000 | 0.978 | 0.488 | 0.592 | 0.546 | -1.492 |
| P23946 | 1.000 | 0.618 | 0.376 | 0.320 | 0.430 | -2.080 |
| O60844 | 1.000 | 1.009 | 0.491 | 0.408 | 0.447 | -1.984 |
| Q86TX2 | 1.000 | 0.996 | 0.570 | 0.541 | 0.557 | -1.445 |
| P04745 | 1.000 | 1.170 | 0.661 | 0.564 | 0.564 | -1.411 |
| P12724 | 1.000 | 1.043 | 0.600 | 0.585 | 0.580 | -1.344 |
| Q16836 | 1.000 | 1.055 | 0.526 | 0.534 | 0.516 | -1.632 |
| O75356 | 1.000 | 1.078 | 0.445 | 0.416 | 0.415 | -2.171 |
| P06865 | 1.000 | 0.968 | 0.597 | 0.609 | 0.613 | -1.207 |
| Q9Y6U3 | 1.000 | 1.106 | 0.650 | 0.532 | 0.561 | -1.425 |
| Q14508 | 1.000 | 1.246 | 0.325 | 0.314 | 0.284 | -3.102 |
| Q13576 | 1.000 | 1.062 | 0.620 | 0.567 | 0.575 | -1.363 |
| Q14002 | 1.000 | 0.614 | 0.192 | 0.304 | 0.307 | -2.911 |
| P18859 | 1.000 | 1.077 | 0.646 | 0.621 | 0.610 | -1.218 |
| O95154 | 1.000 | 1.285 | 0.557 | 0.445 | 0.438 | -2.033 |
| P45954 | 1.000 | 0.805 | 0.530 | 0.471 | 0.555 | -1.452 |
| P50225 | 1.000 | 1.655 | 0.593 | 0.573 | 0.439 | -2.030 |
| Q9HCB6 | 1.000 | 1.272 | 0.482 | 0.528 | 0.445 | -1.999 |
| P09471 | 1.000 | 0.961 | 0.430 | 0.450 | 0.449 | -1.976 |
| Q6UWP2 | 1.000 | 1.020 | 0.433 | 0.536 | 0.479 | -1.813 |
| P04066 | 1.000 | 1.227 | 0.568 | 0.578 | 0.515 | -1.639 |
| O00339 | 1.000 | 1.204 | 0.549 | 0.597 | 0.520 | -1.612 |
| P46108 | 1.000 | 0.942 | 0.551 | 0.500 | 0.541 | -1.514 |
| P07477 | 1.000 | 1.828 | 0.582 | 0.617 | 0.424 | -2.116 |
| P10301 | 1.000 | 1.032 | 0.469 | 0.478 | 0.466 | -1.885 |
| Q9HAT2 | 1.000 | 0.944 | 0.409 | 0.482 | 0.459 | -1.923 |
| Q96DG6 | 1.000 | 1.237 | 0.648 | 0.454 | 0.493 | -1.745 |
| Q96CN7 | 1.000 | 1.144 | 0.396 | 0.634 | 0.480 | -1.810 |
| Q13683 | 1.000 | 0.974 | 0.642 | 0.504 | 0.580 | -1.342 |
| O43181 | 1.000 | 0.914 | 0.389 | 0.600 | 0.517 | -1.628 |
| P16219 | 1.000 | 1.072 | 0.520 | 0.642 | 0.560 | -1.428 |
| P10915 | 1.000 | 0.848 | 0.324 | 0.344 | 0.362 | -2.508 |
| Q7Z7G0 | 1.000 | 0.982 | 0.615 | 0.554 | 0.590 | -1.302 |
| O76038 | 1.000 | 1.977 | 0.662 | 0.500 | 0.391 | -2.319 |
| P09601 | 1.000 | 1.150 | 0.548 | 0.576 | 0.523 | -1.600 |
| Q9NSC7 | 1.000 | 0.915 | 0.447 | 0.458 | 0.473 | -1.848 |
| Q9HCN8 | 1.000 | 1.350 | 0.501 | 0.580 | 0.460 | -1.915 |
| Q9UPN4 | 1.000 | 1.337 | 0.540 | 0.639 | 0.504 | -1.688 |
| P01275 | 1.000 | 2.576 | 0.271 | 0.283 | 0.155 | -4.599 |
| P18827 | 1.000 | 1.001 | 0.626 | 0.566 | 0.596 | -1.277 |
| P20933 | 1.000 | 0.908 | 0.574 | 0.590 | 0.610 | -1.218 |
| O43704 | 1.000 | 1.041 | 0.376 | 0.552 | 0.455 | -1.945 |
| P32004 | 1.000 | 1.146 | 0.596 | 0.616 | 0.565 | -1.408 |
| P10645 | 1.000 | 1.621 | 0.579 | 0.625 | 0.460 | -1.918 |
| P28289 | 1.000 | 0.924 | 0.330 | 0.445 | 0.403 | -2.242 |
| P24043 | 1.000 | 1.086 | 0.613 | 0.591 | 0.577 | -1.355 |
| Q13740 | 1.000 | 0.888 | 0.540 | 0.482 | 0.541 | -1.513 |
| O43570 | 1.000 | 1.121 | 0.582 | 0.657 | 0.584 | -1.325 |
| Q8NFL0 | 1.000 | 1.118 | 0.464 | 0.477 | 0.444 | -2.000 |
| A0AV96 | 1.000 | 1.089 | 0.638 | 0.558 | 0.572 | -1.377 |
| Q6ZMP0 | 1.000 | 0.846 | 0.545 | 0.455 | 0.542 | -1.511 |
| P06870 | 1.000 | 1.250 | 0.567 | 0.561 | 0.501 | -1.703 |
| Q96FZ7 | 1.000 | 1.096 | 0.558 | 0.594 | 0.550 | -1.477 |
| Q96IJ6 | 1.000 | 0.956 | 0.580 | 0.611 | 0.609 | -1.222 |
| P09417 | 1.000 | 0.942 | 0.609 | 0.604 | 0.625 | -1.161 |
| Q5JTB6 | 1.000 | 0.729 | 0.462 | 0.388 | 0.492 | -1.749 |

**IIIC**

| **Accession** | **Relative Abundance in IIIC Group** | | | | **Fold Change** | **log_1.5_(fold change)** |
| --- | --- | --- | --- | --- | --- | --- |
|  | **126-G3** | **128-G4** | **130-P3** | **131-P4** |  |  |
| Q9HCN8 | 1.000 | 1.008 | 1.594 | 1.545 | 1.564 | 1.103 |
| P01860 | 1.000 | 1.117 | 2.773 | 2.066 | 2.285 | 2.038 |
| P07602 | 1.000 | 1.079 | 1.728 | 1.658 | 1.629 | 1.203 |
| P07686 | 1.000 | 1.080 | 1.683 | 1.670 | 1.612 | 1.178 |
| P07305 | 1.000 | 0.823 | 1.622 | 1.852 | 1.906 | 1.591 |
| Q9C075 | 1.000 | 1.064 | 1.617 | 3.313 | 2.388 | 2.147 |
| P83881 | 1.000 | 0.976 | 1.650 | 1.808 | 1.750 | 1.381 |
| Q9GIY3 | 1.000 | 1.056 | 1.615 | 1.843 | 1.682 | 1.283 |
| P05062 | 1.000 | 1.067 | 1.834 | 2.061 | 1.885 | 1.563 |
| O95758 | 1.000 | 1.010 | 1.599 | 1.602 | 1.592 | 1.147 |
| P02741 | 1.000 | 1.027 | 2.262 | 3.403 | 2.794 | 2.534 |
| Q9BS40 | 1.000 | 1.421 | 2.219 | 2.146 | 1.803 | 1.454 |
| Q9P2A4 | 1.000 | 1.106 | 1.735 | 1.868 | 1.710 | 1.324 |
| P35749 | 1.000 | 1.185 | 0.629 | 0.626 | 0.575 | -1.367 |
| P17661 | 1.000 | 0.883 | 0.489 | 0.475 | 0.512 | -1.652 |
| Q9UPN4 | 1.000 | 0.948 | 0.319 | 0.414 | 0.376 | -2.414 |
| P62736 | 1.000 | 0.978 | 0.635 | 0.554 | 0.601 | -1.255 |
| Q01995 | 1.000 | 1.136 | 0.574 | 0.548 | 0.525 | -1.589 |
| P07951 | 1.000 | 1.013 | 0.586 | 0.569 | 0.574 | -1.369 |
| O15061 | 1.000 | 0.949 | 0.626 | 0.560 | 0.609 | -1.225 |
| Q15005 | 1.000 | 0.885 | 0.425 | 0.486 | 0.483 | -1.796 |

**Supplementary Table S4** DEPs expression measurement information.
